# Marine heatwaves alter the nursery function of coastal habitats for juvenile Gulf of Alaska Pacific cod

**DOI:** 10.1038/s41598-024-63897-w

**Published:** 2024-06-27

**Authors:** Hillary L. Thalmann, Benjamin J. Laurel, L. Zoe Almeida, Kaitlyn E. Osborne, Kaylee Marshall, Jessica A. Miller

**Affiliations:** 1 Department of Fisheries, Wildlife, and Conservation Sciences, Oregon State University, Coastal Oregon Marine Experiment Station, Hatfield Marine Science Center, 2030 SE Marine Science Dr., Newport, OR 97365 USA; 2 NOAA Alaska Fisheries Science Center, Hatfield Marine Science Center, 2030 SE Marine Science Dr., Newport, OR 97365 USA

**Keywords:** Fisheries, Marine biology, Climate-change ecology

## Abstract

Marine Heatwaves (MHWs) can directly influence survival of marine fishes, particularly for early life stages, including age-0 juveniles during their residence in coastal nursery habitats. However, the ability of nurseries to support high fish densities, optimize foraging and growth, and protect against predators may be altered during MHWs. Gulf of Alaska Pacific cod (*Gadus macrocephalus*) larval, juvenile, and adult abundances declined dramatically following MHW events in 2014–2016 and 2019. To evaluate coastal nursery function during MHWs, we compared diet composition, recent growth, size, condition, and abundance of age-0 juveniles throughout their first summer before, during, and between MHWs. Diet shifted to larger prey during MHWs, particularly mysids, but diet did not appear to influence growth. We observed faster growth rates during MHWs, yet even when accounting for growth, we could not explain the higher body sizes observed in August during MHWs. Together with lower abundance and the near absence of small fish in the nursery by August during MHWs, these patterns highlight potential for size-selection and a reduced ability of nursery habitats to buffer against environmental variability during MHWs, with only a small number of large “super survivors” persisting through the summer.

## Introduction

Increased frequency and duration of marine heatwaves (MHWs) due to anthropogenic climate change may influence growth and survival of marine fishes around the globe^1,2^.  Anomalous warming associated with MHWs can push ocean temperatures beyond historical thresholds and provide a unique opportunity to examine the acute effects of extreme temperatures on fishes in situ^3^*.* In the past decade, the Gulf of Alaska (GOA) has experienced warming due to two major MHWs. The 2014–2016 Northeast Pacific MHW led to temperature anomalies  greater than 3 °C above the climatological baseline^4^ and was followed by another MHW in 2019 that also produced temperature anomalies greater than  3 °C^5^. Together, these MHWs are considered the most extreme warming events on record in the Northeast Pacific, leading to unprecedented and prolonged shifts in the region’s biological community^6^. During the MHWs, the GOA experienced shifts in phyto- and zooplankton biomass and community structure^7^, declines in abundance and energetic quality of forage fishes^8^, reduced biomass and altered distribution patterns of groundfish^9^, and sharp declines in the abundance of seabirds^10^ and marine mammals^11^.

Warming is often associated with larger body sizes in juvenile ectotherms and a smaller size at maturity^12^. This pattern, known as the ‘Temperature-Size Rule’, is regularly attributed to mechanisms such as faster growth rates^13^ and elevated metabolism^14^ early in life leading to maturation at smaller sizes. However, while increased size and growth rates of juvenile fishes at high temperatures are well-supported in the lab^15,16^, these patterns may be weakened or reversed in the field, where fish experience both the direct and indirect effects of elevated temperatures^17–19^. Increased temperatures can lead to earlier spawn timing for fishes^20^, resulting in fish that are both older and larger when they enter their first winter than conspecifics in cooler conditions. Changes to the foraging environment can alter the type^21^, abundance^7^, and quality^22^ of prey resources, which may hinder an individual’s ability to consume enough food to maintain their growth and body condition. Combined with earlier spawning of larval fishes, changes to the foraging environment may also lead to mis-matches in the seasonal availability of zooplankton to larval fish^23^. Further, the effects of parentage^24^, prior experience^25^, behavior^26^, and selection in the early life^27^ can all contribute to variation in size, growth, and survival in the field. Thus, in the field, larger juvenile sizes at higher temperatures could be due to a myriad of factors in addition to elevated growth.

Size and survival of juvenile fishes reflect their prior growth, foraging, and predation history, which often occurs in coastal habitats, particularly those that function as nurseries in which individuals can successfully forage, grow, avoid predators, and build lipid stores to help them survive their first winter^28,29^. A combination of high productivity and low predation risk for juvenile fishes can lead to increased biomass of smaller individuals, which can serve as a useful measure of nursery function of these habitats^30^. However, nursery function may be altered during MHWs, which may reduce the ability of these habitats to support juvenile fish, particularly smaller individuals. Juvenile fish exhibit ontogenetic variation in temperature tolerance^31^, and smaller individuals typically remain in shallower, warmer waters to avoid predators (e.g. the Shallow Water Refuge Hypothesis^32–34^). This may disadvantage smaller individuals during MHWs, particularly since shallow coastal nurseries are likely to warm more quickly than deeper areas^35^. Changes to juvenile fish abundance have been observed in nearshore habitats across the Northeast Pacific in response to MHWs^36,37^, although these patterns are highly variable, with some species decreasing in abundance while others increased or remained stable. These fluctuations may be driven by several mechanisms, including increased predation risk^38^, reduced abundance and cover of habitat-forming seagrasses and macroalgae^39^, and shifts in the forage base^6^, potentially leading to increased starvation. Other studies have observed increases in fish size during periods of warming in the nursery^35^, potentially due to increased predation on smaller individuals^30,40^, or a reduction of density-dependent effects in the nursery, which may contribute to faster growth rates and larger sizes of surviving individuals, leading to only a few “super survivors” that are larger and in better condition than individuals in years with higher overall survival^41,42^. Understanding whether and to what degree MHWs influence growth and survival of juvenile fishes in nurseries remains a critical knowledge gap.

GOA Pacific cod (*Gadus macrocephalus*) experienced near failure in recruitment during the anomalous MHW conditions in 2014–2016 and 2019, leading to the closure of the federal GOA fishery in 2020^43^. Dramatic declines of Pacific cod larval and age-0 juvenile abundances occurred almost immediately following the onset of MHW conditions, likely due to reduced hatch success^44^ and potential mismatches in prey resources available to first-feeding larvae^23^. Follow-up studies found additional demographic changes in age-0 cohorts during MHWs, including a nearly 3-week shift to earlier hatch dates in the spring^19^, which contributed to predominantly larger and older juveniles entering coastal habitats in early summer^45^. During the MHWs, relatively large body size and low abundance of juvenile Pacific cod were observed post-settlement in coastal habitats across the GOA^46^. Notably, Abookire et al.^46^, demonstrated anomalously low abundance of juvenile Pacific cod during MHWs in both the central and western GOA. However, it remains unknown how well coastal habitats supported juveniles throughout the duration of their first summer in the nursery during MHW conditions.

In this study, we assessed whether GOA coastal habitat provided similar support for age-0 Pacific cod by comparing summer foraging, growth, and survival during recent MHWs (2014–2016, 2019) to years before (2006–2013) and between MHWs (2017–2018). Our analyses relied on collections of juvenile Pacific cod captured in shallow (2-3 m) nursery habitat near Kodiak Island, AK in mid-July, recently after settlement, and in late-August, after approximately two months of nursery residence. We tested the following null hypotheses: during MHWs, age-0 Pacific cod in Kodiak Island coastal nurseries will have (H_1_) similar abundances; (H_2_) similar body sizes and condition; (H_3_) similar stomach fullness and diet composition; and (H_4_) similar growth rates after accounting for covariates of density, body size, and diet composition compared to years before and between MHWs. Further, as expected by the proposed mechanisms of the Temperature-Size Rule, (H_5_) nursery growth rates will explain size differences between fish captured in July and August across all years of the study.

## Results

### Marine heatwave classification

The widely observed GOA MHWs were manifested in the nearshore waters of Kodiak Island, which experienced extreme and prolonged heatwave conditions in 2014, 2015, 2016, and 2019. In these years, heatwave conditions occurred during 1080 of 1461 days (see Supplementary Table S1). Based on these classifications, we binned years into three categories: ‘Before Heatwave’ (2006–2013); ‘Heatwave’ (2014–2016, 2019); and ‘Between Heatwave’ (2017–2018). We identified the 2 years between the MHWs (2017–2018) as distinct from conditions before the MHWs due to warmer ocean temperatures and larger fish sizes compared to those observed before the MHWs. While heatwave designations were useful to examine broad patterns, we also used daily temperature data from coastal Kodiak Island to understand thermal variability at a finer scale (see Supplementary Fig. S1). Highest temperatures were in 2019, with mean August temperatures reaching 13.10 °C. Lowest temperatures were present in July 2008, with mean temperatures reaching only 7.92 °C.

### Abundance

**Figure 1:**
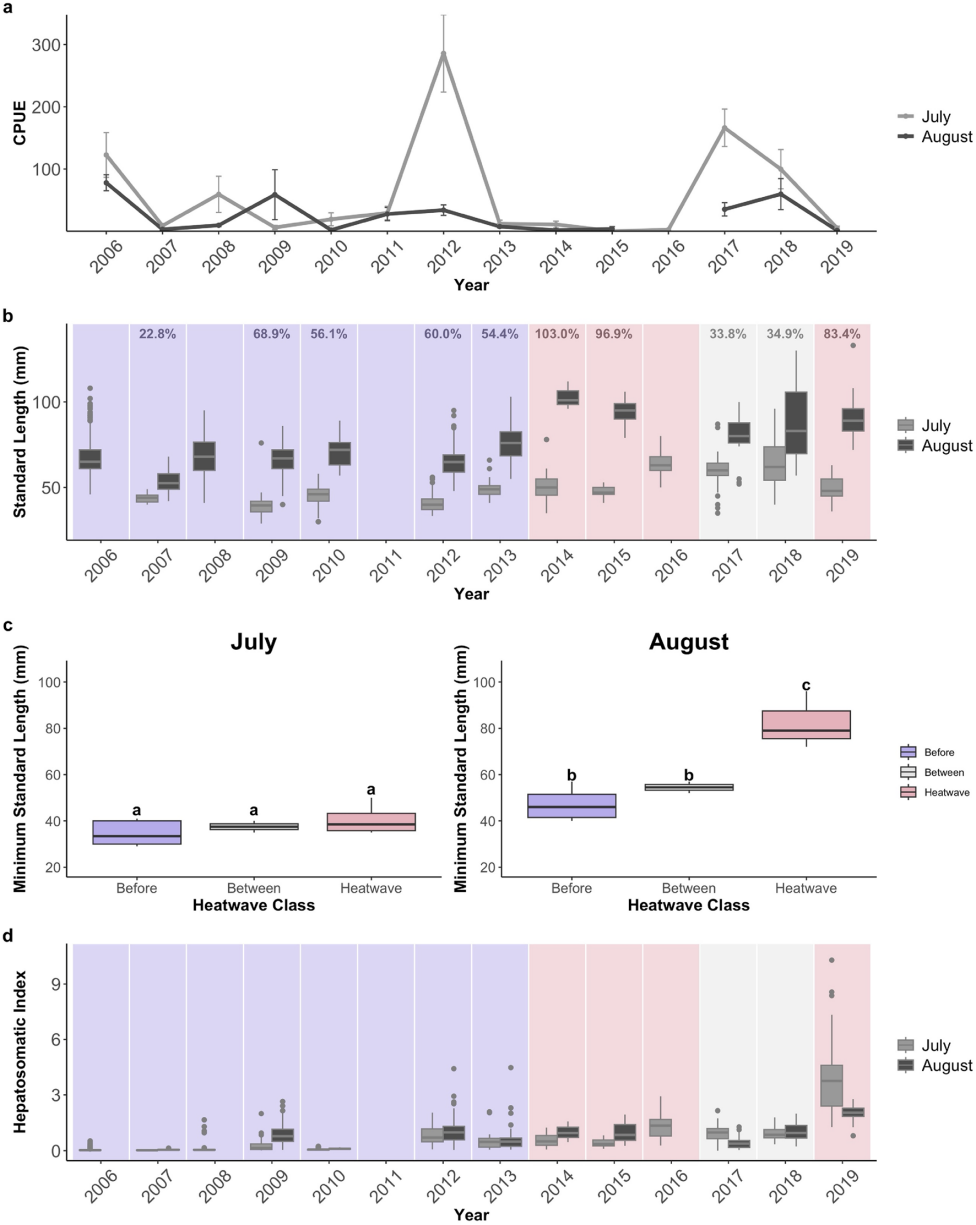
(**a**) Juvenile Pacific cod catch per unit effort (CPUE) from Kodiak Island nursery habitats declined during MHWs in 2014–2016 and 2019. (**b**) Standard length increased between July and August during MHWs, and (**c**) minimum standard length increased by August during MHWs, with no individuals smaller than 72 mm present in sampling. (**d**) Hepatosomatic index was highly variable, with particularly good condition observed in 2019. Percentages for panel (**b**) represent the mean percent increase between July and August standard length for each year. Plots are shaded based on their heatwave class, with blue representing years before the MHWs, red representing years during MHWs, and light grey representing years between MHWs. Error bars for panel a represent standard error. Boxplots extend from the first to the third quartiles of the data, with whiskers that extend to the largest values no further than the 1.5 × IQR. “a” and “b” notation for panel c represents significance based on a linear model for July and August predicting minimum standard length by heatwave class.

Between 2006 and 2019, 1420 juvenile Pacific cod were collected during annual beach seine surveys in nearshore nursery habitats near Kodiak Island, AK. Sampling was conducted twice per summer to encapsulate patterns in early settlement in mid-July and post-settlement approximately 6 weeks later at the end of August. Subsets of these fish were selected for diet composition (n = 525) and growth analyses (n = 488) via random selection ensuring representation across the size range (Table 1; for this information by year, see Supplementary Table S2). During MHWs, juvenile annual mean abundance declined by 92.2% and 95.9%, respectively, compared to years before and between MHWs (Fig. 1a). However, we did not observe significant differences in abundance across months and heatwave classes when year was included as a random variable (see Supplementary Table S3).

**Table 1:** Sample sizes for total catch, diet composition, and growth analyses of juvenile Pacific cod captured in July and August in years before, during, and between marine heatwaves near Kodiak Island, AK. Summary statistics are reported for Trident Bay, AK temperature (°C), age-0 Pacific cod catch per unit effort (CPUE), standard length (mm), and body mass (g). Summary statistics are reported as mean ± standard error.

Sampling month	Heatwave class	Years	Total *n*	Diet *n*	Growth *n*	Trident Bay mean temp. (°C)	CPUE	Standard length (mm)	Body mass (g)
July	Before	2007, 2009, 2010, 2012, 2013	315	125	137	8.76 ± 0.04	63.13 ± 10.42	43.4 ± 0.4	0.86 ± 0.03
	Heatwave	2014, 2015, 2016, 2019	128	84	69	10.90 ± 0.08	4.71 ± 1.60	55.1 ± 0.8	1.84 ± 0.09
	Between	2017, 2018	78	51	44	9.42 ± 0.05	132.94 ± 22.01	62.7 ± 1.5	2.85 ± 0.22
August	Before	2006, 2007, 2008, 2009, 2010, 2012, 2013	777	162	144	9.70 ± 0.03	31.69 ± 6.13	67.1 ± 0.4	3.11 ± 0.07
	Heatwave	2014, 2015, 2019	56	53	50	12.10 ± 0.08	2.13 ± 0.99	92.7 ± 1.5	9.62 ± 0.61
	Between	2017, 2018	66	50	44	10.20 ± 0.04	47.45 ± 13.54	84.0 ± 2.2	7.08 ± 0.63

### Size and condition data

Variation in standard length and body mass (n = 1420) were related to the interaction between heatwave class and month with year as a random factor (Standard Length: R^2^_marginal_ = 0.61; R^2^_conditional_ = 0.71; Body Mass: R^2^_marginal_ = 0.56; R^2^_conditional_ = 0.73). The increase in juvenile body size between July and August was notably greater during MHWs when fish size-at-capture nearly doubled (see Supplementary Table S3). We observed a 94.5 ± 5.8% increase in mean fish length between July and August during MHWs that was not matched in other years (Fig. 1b). Before the heatwaves, mean length increased by 52.5 ± 7.8% between July and August, and between the heatwaves, length increased by 34.3 ± 0.6%. In addition, we observed a significant increase in minimum standard length by August during MHWs that was not observed in other years, with fish smaller than 72 mm absent in August samples during MHWs (Linear Regression, P = 0.0001). We did not detect this increase in minimum standard length in July during MHWs (Linear Regression, P = 0.18), which suggests that, by August of MHW years, smaller-bodied individuals were nearly absent in the nursery (Fig. 1c). These patterns were magnified in fish body mass, with a 725.6 ± 80.2% increase in mean fish body mass between July and August during MHWs, compared to a 288.4 ± 54.0% increase before MHWs and a 146.4 ± 23.3% increase between MHWs (see Supplementary Fig. S2).

Body condition was highly variable across heatwave classes. Variation in hepatosomatic index (HSI) was explained by an interaction between heatwave class and month with year as a random factor (R^2^_marginal_ = 0.15; R^2^_conditional_ = 0.59). HSI was higher during MHWs and in August for most years (Fig. 1d). However, this pattern was strongly influenced by high body condition in 2019, particularly in July, with HSI values more than 3 times higher than in other years. When the model was run without 2019 values, we still observed higher HSI values during MHWs, but there was no longer an interaction between heatwave class and month (see Supplementary Table S3). Month and heatwave class had no influence on length–weight condition residuals (R^2^_marginal_ = 0.05; R^2^_conditional_ = 0.78). However, condition was poor in 2007 and 2008, which were the two coldest years of the study, with fish 99.4% lighter weight at size than fish from any other years (see Supplementary Fig. S2).

### Diet composition analysis

Across all years, stomach contents averaged 1.8% of the fish’s overall body weight. Only 2.3% of fish had empty stomachs (n = 12). Most of these empty stomachs occurred in July (75%); however, we did not observe any clear patterns with year or heatwave class (see Supplementary Fig. S3). Stomach fullness was influenced by a significant interaction between Month and Heatwave class (see Supplementary Table S3). Before MHWs, stomach fullness was 25.8 ± 9.1% higher in August than in July, but during and between MHWs, stomach fullness was comparable across months.

**Figure 2:**
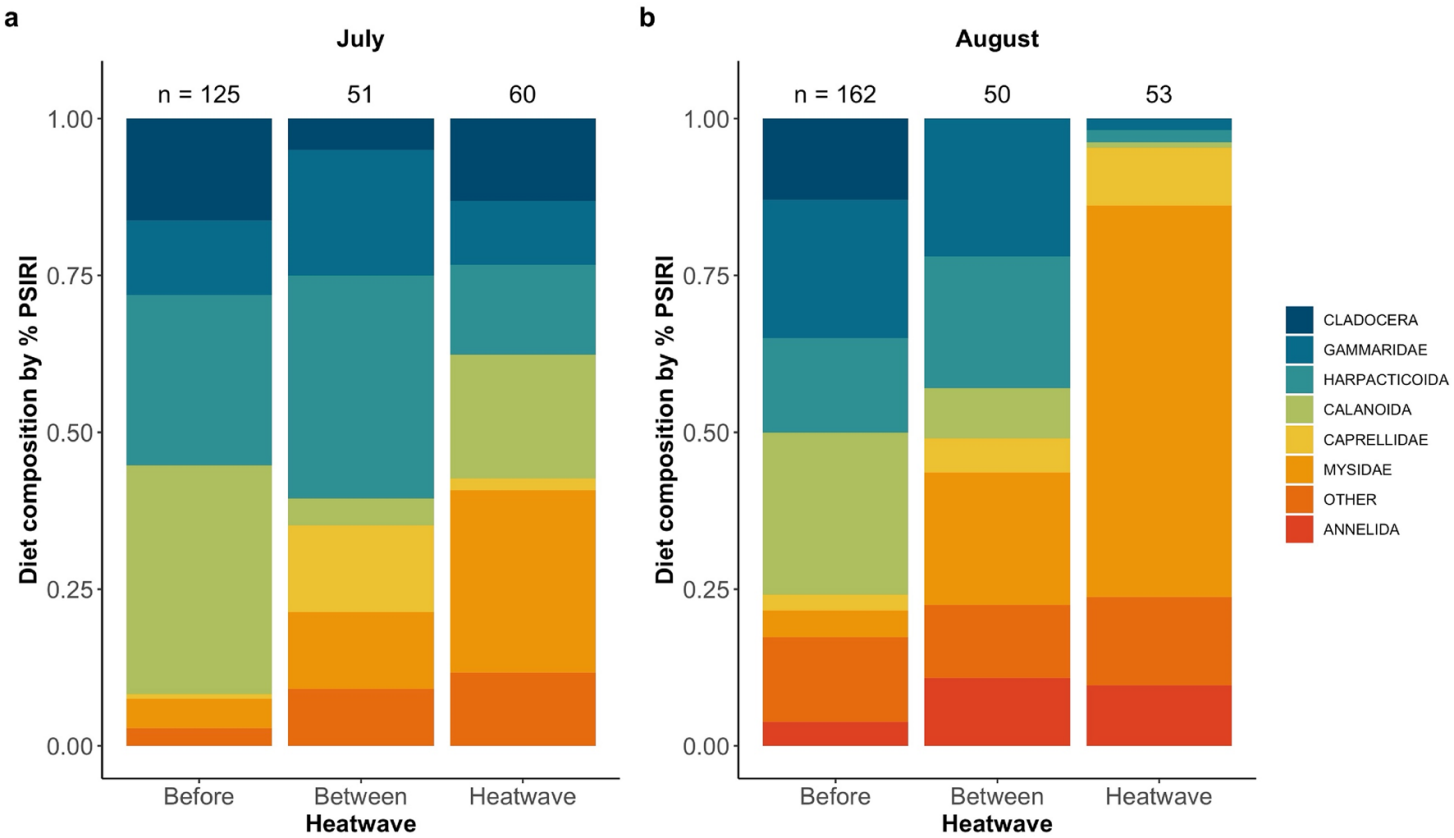
Juvenile Pacific cod diet composition varied during MHWs, with mysids (represented in orange) representing a large portion of the diet. Diet composition is represented by percent Prey Specific Index of Relative Importance (PSIRI) between 2006 and 2019 for July (left panel) and August (right panel). The “other prey” category includes all prey that was not found in at least 3.5% of all stomachs sampled.

Pacific cod diet composition was significantly different across heatwave classes for both months (Fig. 2). In July, mysids were the most common prey species during MHWs, representing 29.1% of the total diet, compared to 4.7% before MHWs and 12.2% between MHWs (MRPP; *A* = 0.072; *P* = 0.001). Caprellid amphipods were associated with July diet composition between MHWs, while calanoid copepods were characteristic of years before and during MHWs. By August, mysids remained the most common prey item during MHWs, were present in more than 75% of all sampled stomachs, and represented 62.4% of the total diet, compared to 4.3% before the MHWs and 21.2% between MHWs (MRPP; A = 0.074; P = 0.001). Before the MHWs, August fish consumed smaller species, particularly cladocerans, but this taxon was virtually absent from diets during and between MHWs. August diet between MHWs shared characteristics with diets from both before and during MHWs. Indicator species for years before and between MHWs included gammarid amphipods, harpacticoid copepods, and small calanoid copepods, while indicator species for years during and between MHWs included caprellid amphipods and annelid worms. To see diet information by year, please see Supplementary Fig. S4.

**Figure 3:**
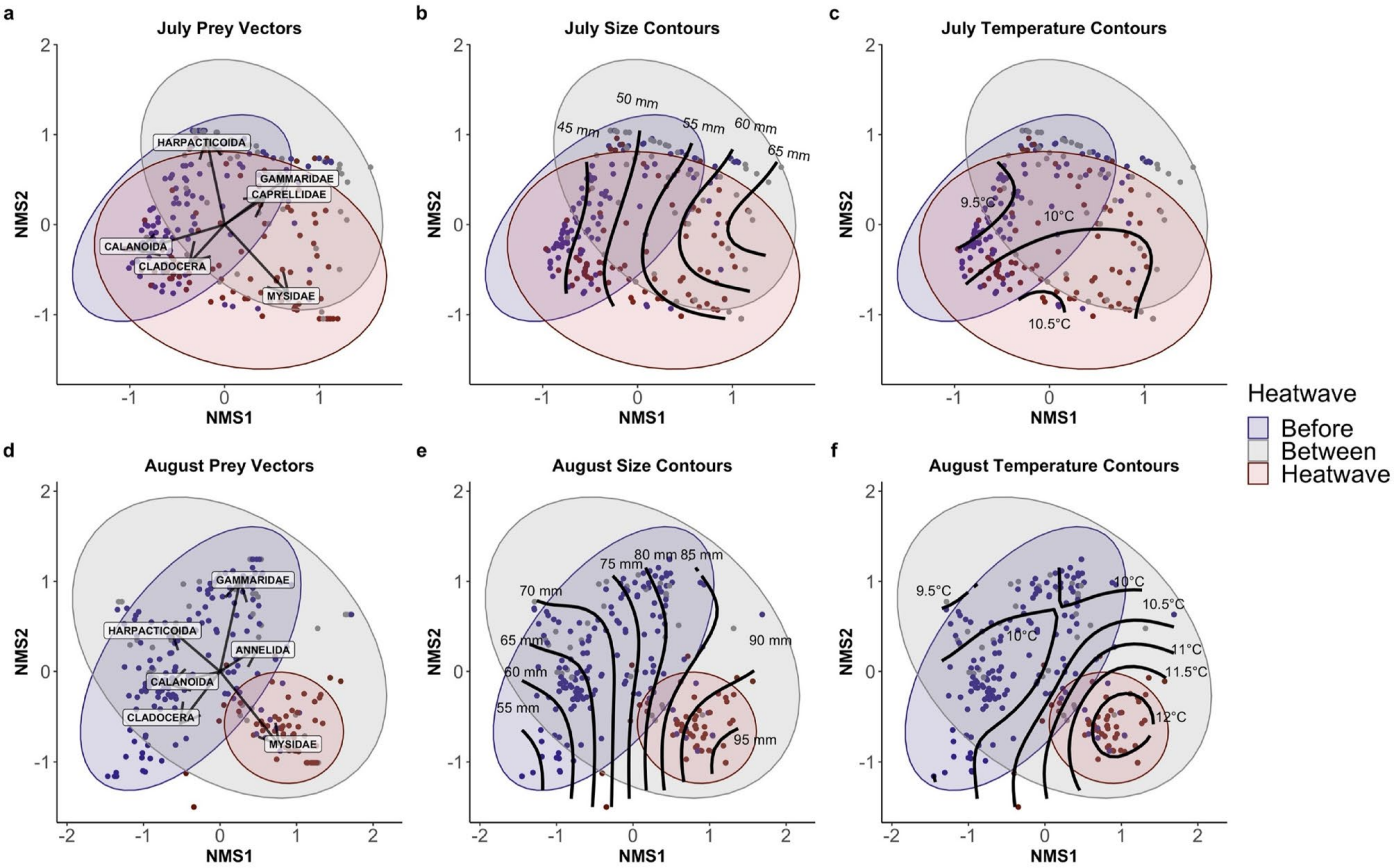
Nonmetric multidimensional scaling (NMS) biplot of July (top) and August (bottom) diet composition showed strong separation in juvenile Pacific cod diet composition during MHWs, particularly by August. For both July and August, fish size was correlated with axis 1 of the ordination, and by August, temperature was well correlated with axis 1 of the ordination. Ellipses represent 95% confidence intervals in the two major axes and are shaded based on their heatwave class, with blue representing years before the MHWs, red representing years during MHWs, and light grey representing years between MHWs. Prey vectors (**a**,**d**) indicate significant prey species correlations (P < 0.05) with the two major axes. Standard length contours (**b**,**e**) and Trident Bay temperature contours (**c**,**f**) indicate the size of juvenile Pacific cod and the mean daily temperature in Trident Bay, respectively, in relation to Axes 1 and 2 of the NMS ordination.

NMS ordinations stabilized on 3-dimensional solutions (July: final stress = 0.117; August: final stress = 0.123) and showed patterns between diet composition and fish size, body condition, and temperature across both months of the analysis (Fig. 3). In July, fish size was correlated with Axis 1 scores (r = 0.56), while body condition and temperature were not correlated with any axis (r < 0.2). By August, size, condition, and temperature were all positively correlated with Axis 1 of the ordination (SL: r = 0.66; LW Condition: r = 0.50; HSI: r = 0.46; Temperature: r = 0.53). Larger fish were more commonly associated with larger prey items such as mysids and annelids, and these patterns were well correlated with higher temperatures. However, ordinations that were restricted to fish of sizes between 50–60 mm in July and 70–90 mm in August yielded similar results as the larger dataset (see Supplementary Fig. S5). Even among individuals of similar sizes, diet composition differed across heatwave classifications in both July (MRPP; *A* = 0.119; *P* = 0.001) and August (*A* = 0.239; *P* = 0.001).

### Growth analysis

**Figure 4:**
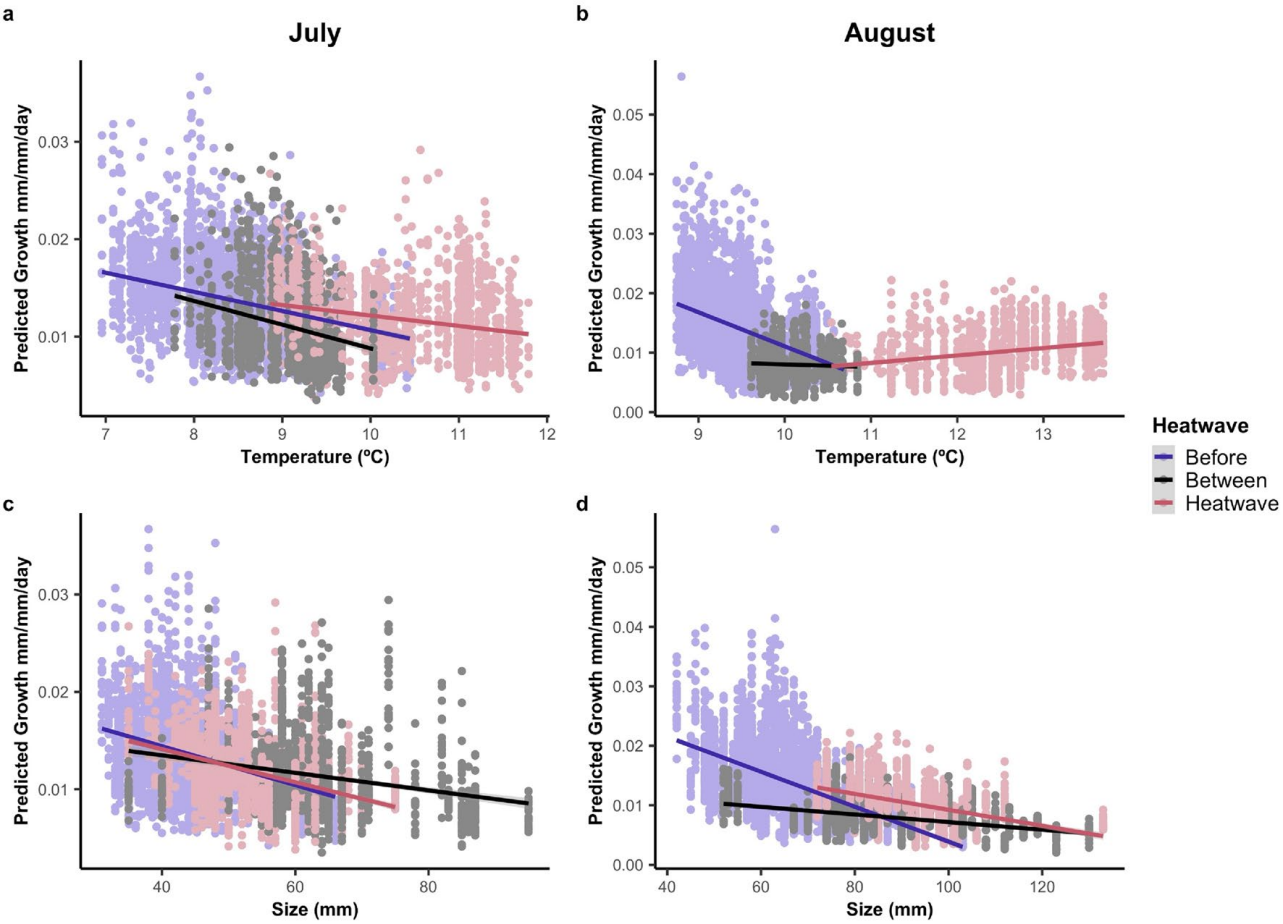
Predicted relative growth (mm/mm/day) for juvenile Pacific cod declined with size and temperature across most heatwave classes in July (top) and August (bottom). These plots show predicted relative growth rates with 95% confidence intervals based on linear mixed effects models fitted separately for July (left) and August (right) from 2006 to 2019 against the actual size and temperature data for both months. Individual data points are shaded based on their heatwave class, with blue representing years before the MHWs, red representing years during MHWs, and light grey representing years between MHWs.

Growth was significantly faster during MHWs compared to before and between them after accounting for covariates. For both months, predicted growth rates were faster during the MHWs and explained by size, temperature, and heatwave class. As temperatures increased, predicted growth rates declined or plateaued across all months and years (Fig. 4). In general, juveniles grew faster in cooler water and at smaller sizes within heatwave classes, and these patterns were most prevalent in years before the MHWs. However, due to large increases in body size and water temperature during the MHWs, there was limited overlap in these variables, which was particularly evident by August when small fish (< 72 mm) were largely absent from sampling. Diet composition, stomach fullness, and abundance did not significantly explain growth variation in either month (Table 2).

**Table 2:** Linear mixed effects model results for the response of July and August relative growth rates to size, temperature, and heatwave class.

Month	Fixed effect	Estimate	Std. error	df	*t-*value	*p-*value
July	Intercept	− 4.43	0.023	4999	− 196.81	< 0.0001
	Size	− 0.12	0.020	246	− 6.02	< 0.0001
	Temperature	− 0.11	0.010	4999	− 11.34	< 0.0001
	Between MHW	0.07	0.053	246	1.31	0.191
	During MHW	0.15	0.040	246	3.83	0.0002
August	Intercept	− 4.67	0.035	4754	− 133.64	< 0.0001
	Size	− 0.29	0.043	232	− 6.70	< 0.0001
	Temperature	− 0.05	0.039	4754	− 1.39	0.166
	Between MHW	− 0.14	0.059	232	− 2.43	0.016
	During MHW	0.34	0.149	232	2.27	0.024
	Size × Temp	0.15	0.046	4754	3.29	0.001
	Size × MHW (between)	0.13	0.057	232	2.33	0.021
	Size × MHW (during)	− 0.02	0.124	232	− 0.20	0.841
	Temp × MHW (between)	0.14	0.059	4754	2.41	0.016
	Temp × MHW (during)	− 0.01	0.071	4754	− 0.09	0.930
	Size × Temp × MHW (between)	− 0.15	0.056	4754	− 2.75	0.006
	Size × Temp × MHW (during)	− 0.11	0.067	4754	− 1.71	0.088

**Figure 5:**
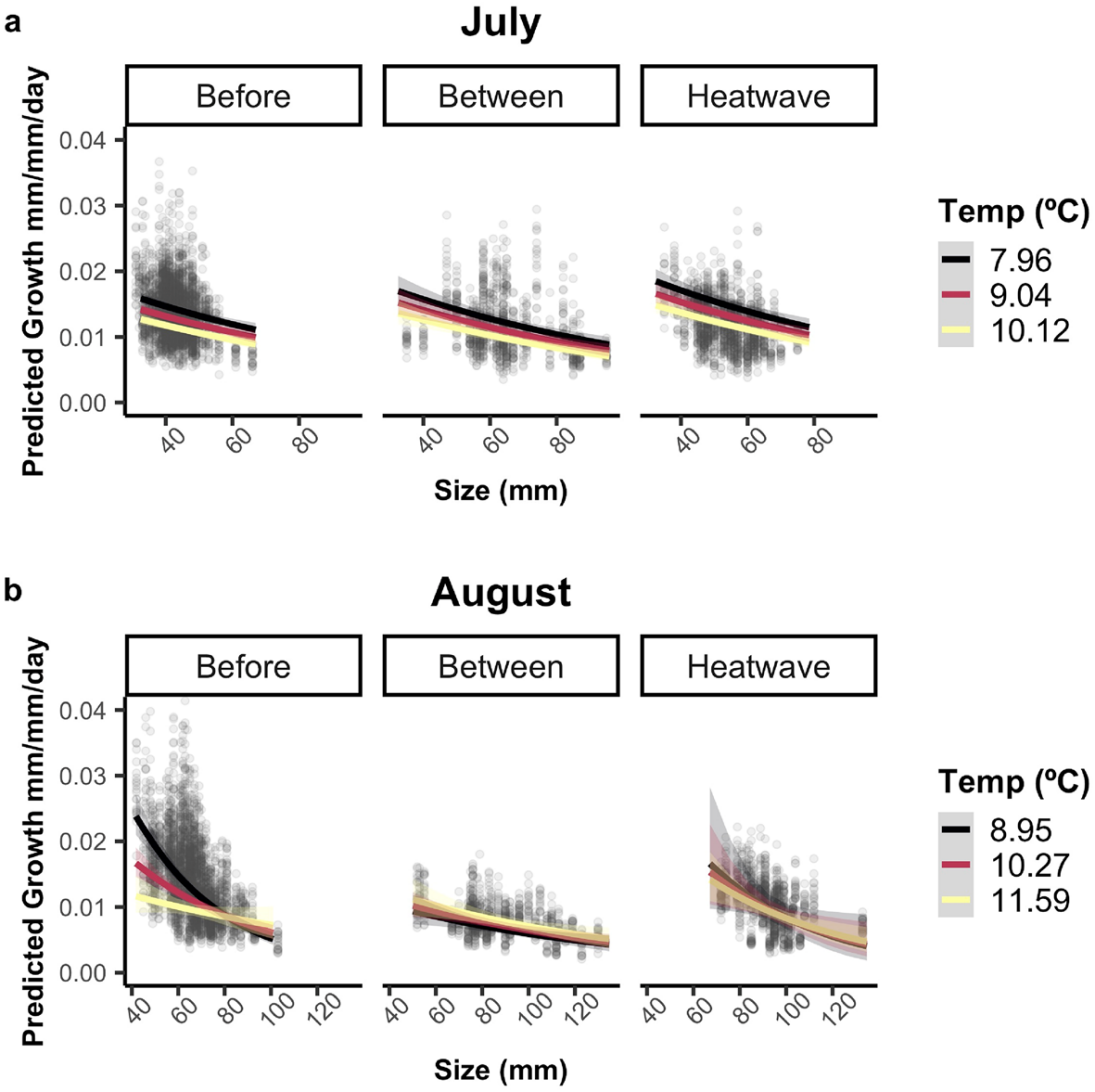
Marginal mean effects of predicted relative growth rate (mm/mm/day) show elevated growth rates during MHWs in both July (top) and August (bottom). Growth rates declined with size and temperature across all months and heatwave classes, although there was limited overlap in temperature and size across all three heatwave classes in August. Marginal means are based on results of linear mixed effects models for each month, and all values are adjusted to otolith increment number 11, which represents the midpoint of our 3-week growth period.

For July growth, the most parsimonious model included the fixed effects of size, temperature, and heatwave class, a random intercept of fish ID, a random slope for day of life, and a first order autocorrelation process (R^2^_marginal_ = 0.210; R^2^_conditional_ = 0.691; Table 2). The effects of size and temperature on growth were consistently negative within all three MHW classes, with smaller fish in cooler temperatures achieving the fastest marginal mean growth rates regardless of heatwave status. However, after accounting for relationships with size and temperature, marginal mean growth rates were ~ 15% faster during MHWs and ~ 7% faster between MHWs compared to growth before the MHWs (Fig. 5a).

Growth patterns became more complicated by August, with the most parsimonious model including the fixed effects of size, temperature, heatwave class, and all interactions, a random intercept of fish ID, a random slope for day of life, and a first order autocorrelation process. The fixed effects in the August model explained nearly two times the growth variation than the July model (R^2^_marginal_ = 0.424; R^2^_conditional_ = 0.861; Table 2), with a 3-way interaction between size, temperature, and heatwave class. During MHWs, August marginal mean growth rates were consistently ~ 33% faster than during other heatwave classes (Fig. 5b). Before MHWs, faster marginal mean growth occurred at smaller sizes; however, the effect of temperature on growth rate diminished as fish size increased. Growth was slowest between MHWs, when fish were only observed within a narrow temperature range and showed a weakly negative relationship with size.

### August size predictions

**Figure 6:**
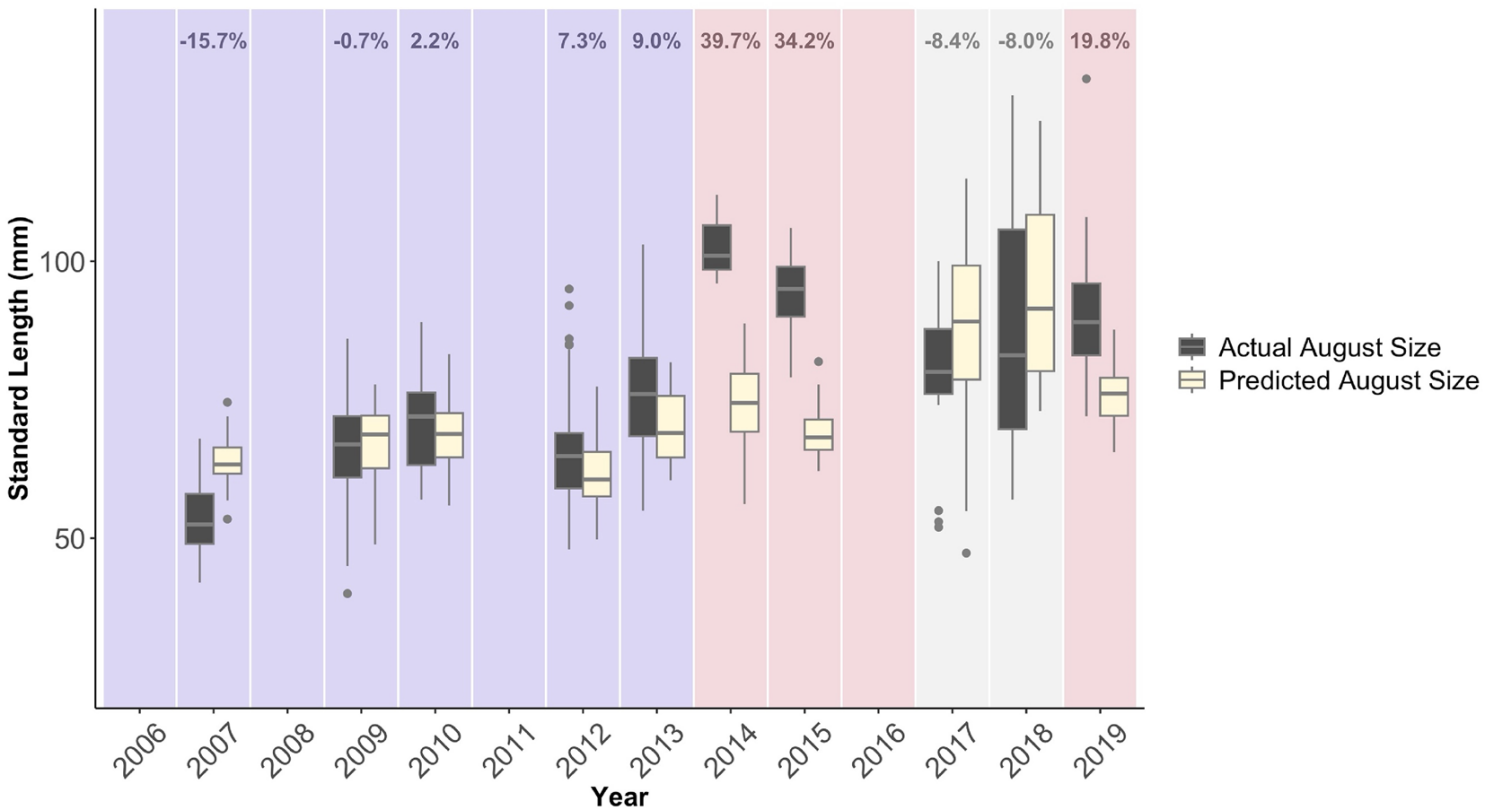
August size during MHWs could not be predicted from growth rates alone. Predicted August sizes were based on July growth rates and calculated by summing mean July growth rates for that year over the number of days between July and August sampling in that year. Percentages represent the mean percent increase between predicted August growth and actual August growth for each year. Dark-grey plots represent actual August sizes and beige-colored plots represent predicted August sizes. Plots are shaded based on their heatwave class, with blue representing years before the MHWs, red representing years during MHWs, and grey representing years between MHWs. Boxplots extend from the first to the third quartiles of the data, with whiskers that extend to the largest values no further than the 1.5 × IQR. Individually plotted points represent outliers beyond the 1.5 × IQR range.

The large increase in body size observed during MHWs between individuals captured in July and August could only partially be explained by elevated growth rates (Fig. 6). During MHWs, the actual size of the August fish was 92.66 mm, 95% CI [89.78, 95.54], which was 31.2 ± 5.6% larger than their expected sizes of 73.81 mm, 95% CI [71.84, 75.78] if July fish continued growing at their observed growth rates (R^2^_marginal_ = 0.416; R^2^_conditional_ = 0.514, see Supplementary Table S4). In contrast, before the MHWs and between the MHWs, the actual sizes of August fish were comparable to, or slightly smaller than, their expected size if July fish continued growing at observed growth rates (Before: 0.4 ± 4.4%; Between: − 8.2 ± 0.2%). However, predicted August sizes aligned with actual August sizes if only largest 15% of July individuals grew to their predicted August sizes (predicted August size of the largest 15% of July individuals: 90.53 mm, 95% CI [87.67, 93.39]).

Simulated size distributions showed similar results as the observed data (see Supplementary Fig. S6). A random draw of samples from this large, simulated population yielded actual August sizes of 93.50 mm, 95% CI [90.30, 96.70], compared to predicted August sizes of 80.60 mm, 95% CI [78.03, 83.17]. In this simulated population, predicted August sizes aligned with actual August sizes if only the largest 25% of July individuals survived to August (predicted August size of the largest 25% of the July individuals from the simulated population: 93.82 mm, 95% CI [92.34, 95.30]).

## Discussion

We provide a comprehensive examination of the impacts of recent MHWs on juvenile Pacific cod throughout their first summer in Gulf of Alaska coastal nursery habitats. Nurseries appeared to meet the presumed increased energetic demands of surviving individuals during MHWs, as indicated by their moderately faster growth in both July and August compared to other years. However, increased growth rates could not explain the large intra-annual differences in fish size observed between July and August during MHWs or the absence of smaller fish (< 72 mm SL) by August in those years. Alternatively, these patterns could be attributed to factors such as increased size-selective mortality and a reduced ability of nurseries to support smaller individuals during MHWs. Individuals captured by late summer during MHWs were likely the “super survivors” of their cohorts, representing only a limited number of large-bodied individuals that were able to forage, grow, and evade predators most successfully^40–42^. Together, these results suggest that the coastal nursery residence could represent an emerging critical period for Pacific cod with continued regional warming.

Intra-annual size-at-capture increased dramatically between July and August during MHW years, and while juveniles exhibited faster growth rates, growth could only account for a portion of the observed size shift, with actual size in August ~ 30% greater than size predicted from growth rate alone. However, we were able to account for the large sizes observed in August if only the largest 15–25% of individuals captured in July grew to their predicted August sizes. These patterns suggest that the increased size of juveniles by August during MHWs was likely driven by size-selective mortality^47^ in the nursery as opposed to faster growth rates during warm conditions. A potential explanation for this apparent increase in size-selective mortality during MHWs is that smaller fish are more sensitive to environmental variability^31^, more susceptible to starvation^48^, or more vulnerable to predators^40,42^, and thus experienced higher mortality rates in the nursery compared to larger fish, particularly by late summer. Larger “super survivors” may have been more common in August sampling due to their ability to select larger and higher quality prey^49^; evade predators^50^; and out-compete smaller conspecifics^51^. While we did not find strong evidence for food-limitation in the nursery during MHWs, it also remains unclear whether sufficient smaller-sized prey resources were available to support smaller fish during MHWs. Overall, the reduction of small-sized fish (< 72 mm) in August during MHWs suggests that the ability of coastal habitats to function as nurseries may have been reduced during these periods of extreme warming^30^.

Alternative explanations for the large increases in size between July and August during MHWs include immigration or emigration of individuals in Kodiak nursery habitats or behavioral shifts during the nursery residence. Unlike Atlantic cod, which settle to the nearshore in multiple pulses^52^, juvenile GOA Pacific cod arrive to coastal nursery habitats in a single pulse in June^53^, and it is unlikely that a new group of juvenile Pacific cod immigrated to the nursery between July and August sampling. Elevated temperatures could lead to behavioral shifts or movement of individuals to deeper, cooler waters (e.g. behavioral thermal regulation^54^), but small fish (not large fish) generally remain in shallow water (e.g. The Shallow Water Refuge Hypothesis^32– 34^) since larger predatory individuals tend to increase with depth (Heincke’s Law^55^). Increased predation risk during the MHWs would likely lead to increased, rather than decreased, numbers of small individuals sheltering in the shallow (< 2 m) beach-seine accessible habitats. In addition, where movement and depth preferences have been investigated, it is typically the larger juveniles (> 100 mm TL) that first move to deeper waters in the fall, making them comparatively less available to the beach seine than smaller individuals, which remain in shallow areas longer^53,56^. However, we observed the opposite pattern, with more large fish and fewer small fish in the beach seines in August during the MHWs. Therefore, the unexpected increase in fish size by August during MHWs is unlikely the result of behavioral changes in the cohort that would make larger fish more available to the gear.

An increase in juvenile fish body size during the MHWs is consistent with the Temperature-Size Rule^12^; however, the mechanisms underpinning the Temperature-Size Rule in fishes remain ambiguous^13^. While large sizes of juvenile fish in warm conditions are often attributed to elevated growth or increased metabolism, we demonstrate that only a portion of the large increase in juvenile Pacific cod size during MHWs could be explained by faster growth in the nursery. Further, in direct contrast to the Temperature-Size Rule, our growth rates exhibited a negative relationship with water temperature. Instead, the larger body size during MHWs were predominantly a result of older ages due to shifting phenology^19^ combined with modestly faster growth, and potentially other factors, including size-selective mortality^47^. It is also unlikely that decoupling between daily otolith increment formation and somatic growth contributed to higher than predicted sizes by August. In some cases of starvation, the otolith can continue to grow even if fish size does not increase^57^. However, if this were the case, we would have overestimated, rather than underestimated, predicted fish sizes in August. These results indicate that factors other than growth rate may better explain changes in size distributions of wild fishes during periods of extreme warming.

Diet composition and stomach fullness did not contribute to Pacific cod growth models in either month despite marked shifts in diet composition during MHWs. Other studies have found strong correlations between gut content and growth in early life stages of fish in the field^58^, but these correlations can be less pronounced at an individual level and can vary across species, life stages, and life history strategies. Before the MHWs, we observed a modest increase in stomach fullness between July and August, but this pattern diminished during and between MHWs, with little change in stomach fullness over the summer. While empty stomachs were present in only 2.3% of all sampled individuals across all heatwave classes, our sampling effort represented only individuals that survived to capture. Individuals that were unable to meet energetic demand likely did not survive, regardless of heatwave class. During the MHWs, the consequences of missing a meal may have been higher than in cooler conditions, particularly for smaller fish as these individuals often exhibit higher metabolic rates and lower lipid storage than larger individuals^48,59^. Increased starvation of small individuals during the MHWs due to shifts in prey availability or increased competition for prey resources may have contributed to the absence of these small fish in sampling efforts by the end of the summer.

Mysids dominated juvenile Pacific cod diet composition during the MHWs, and, in August 2015 and 2019, they were present in more than 75% of all individual fish stomachs. Although coastal GOA zooplankton communities are poorly characterized relative to the pelagic GOA^60,61^, our observed shifts in diet composition during MHWs may be reflective of changes to the nearshore given that Pacific cod are considered opportunistic predators^62^. In the southern California Current, mysid abundance increased during the 2014–2016 MHW^63^, and the similar increase of mysids in Pacific cod diet composition observed during GOA MHWs may be due to changes in their spatial, temporal, or depth distributions that increased their accessibility to juvenile Pacific cod. *Neomysis kadiakensis* and *Exacanthomysis arctopacifica,* the two most abundant species of mysids consumed in this study, aggregate in large groups that migrate between the benthos and the water column^64^, and their distributions may be influenced by stratification in the water column and shifts in nearshore circulation patterns^65^. During the MHWs, increased stratification and altered freshwater runoff^66^; shifts in nearshore macroalgal and substrate composition^39^; and changes to circulation patterns^67^ could have influenced the type, timing, and abundance of these and other nearshore species available to juvenile Pacific cod during the MHWs.

In addition to shifts in prey availability in the nearshore, changes in Pacific cod behavior an ontogeny during MHWs may have further influenced the type and abundance of prey items consumed, although diet shifts persisted even when analysis was limited to fish of similar sizes. Larger prey items such as mysids may have provided an energetic advantage to juveniles, particularly in warm conditions where their metabolism was presumably elevated^43^. Energetic values (kJ/g) of mysids range from 30 to 90% higher than those of gammarid amphipods^68^, a common prey item prior to MHWs, and Pacific cod may have eaten them more often than other potential prey items during the MHWs. For juvenile walleye pollock, increased consumption of higher quality prey can lead to increased body condition, potentially leading to increased overwinter survival^69^. We observed higher body condition for juvenile Pacific cod by August during MHWs, particularly in August 2019, where HSI values were more than three times higher than in other years, a result also observed elsewhere across the GOA^46^. Further, HSI was correlated with Axis 1 of our NMS ordination by August during MHW years. These patterns suggest that surviving juveniles likely had an energetic advantage compared to other years as they entered their first winter during MHWs, although it remains unclear whether this apparent advantage influenced age-1 recruitment the following year.

Despite juveniles being larger and faster-growing, abundance was extremely low during the MHWs, both at settlement and throughout the summer. Annual recruitment of age-0 juveniles is largely determined by spawning dynamics^44^ and larval processes^23^ prior to settlement into the nursery, and anomalously low abundances of larval Pacific cod were observed in GOA ichthyoplankton surveys during MHWs^70^. Our results suggest that annual recruitment in these years was further influenced by size-selective mortality the first summer in the nursery. However, we did not observe high nursery mortality rates between July and August based on raw CPUE from beach seines during MHWs compared to years before and between them. This may be due to a differing mortality structure in the nursery during MHWs. We observed greatest absolute declines in intra-annual nursery abundance in years when juveniles arrived in higher numbers, such as 2012. In MHW years, where low abundances occurred in both months, mortality between July and August may have been more strongly size selective, with high mortality concentrated in the smaller size classes, but comparable overall mortality rates to years before and between MHWs, where mortality rates may have been more evenly distributed across all size classes. In addition, while July CPUE appears to be a robust estimate of relative abundance, August CPUE may not be ideal for estimating nursery-specific mortality rates given patchy density structure and relatively low catches.

In the GOA ecosystem, MHW events are likely to become longer and more frequent due to anthropogenic climate change^1^, and Pacific cod will need to either adapt to these novel conditions or migrate to new environments with more suitable thermal habitat^71,72^. Since the 1990s, southern populations of Pacific cod in the Pacific Northwest and British Columbia have experienced steep declines^45^, and similar patterns in the GOA population may suggest that a phenomenon analogous to winter mortality could become more common with long-term and acute warming^73^. During the MHWs, we observed patterns in juvenile Pacific cod size, diet composition, and growth that were distinct from other years in our analysis, suggesting broad similarities across years associated with MHWs. However, even within our MHW classifications, we observed yearly variability within Pacific cod’s response to MHWs. For example, in 2019, we observed dramatically higher body condition than in other years, with condition in July 2019 more than three times higher than condition in any other year. The 2019 MHW, while shorter than the 2014–2016 MHW, was associated with the warmest temperatures in the Kodiak Island nearshore of the entire study period, with peak temperatures approaching 14 °C. In addition, the 2019 MHW followed several years of persistent warming, and Pacific cod may have exhibited some adaptive capacity by 2019, particularly since some of the juvenile Pacific cod arriving in the nurseries in 2019 may have been the offspring of individuals who survived the first years of the MHWs^25^.

Pacific cod are considered intermediate life history strategists with relatively short life spans and fast growth rates, which may increase their sensitivity to MHWs but could also allow their populations to recover quickly^43,74^. During the years between the MHWs, juvenile Pacific cod CPUE was among the highest we observed with greater demographic variation, including some of the broadest size distributions and slowest growing individuals. These trends may reflect persistent early spawning after the MHWs^19^, selective pressure for specific traits from the previous MHW years^25^, a shift in the parental stock contribution of Kodiak Island juveniles following the MHWs^75^, or a combination of these factors. Improved conditions in the nursery between the MHWs in 2017 and 2018 may illustrate some resilience of the species to periods of anomalous warming^76^; however, less is known about how these cohorts fared through their first winter and beyond.

## Conclusion

We provide evidence for altered nursery function of coastal habitats for juvenile GOA Pacific cod during recent MHWs. Nursery habitats were unable to support small-bodied individuals during recent MHWs, likely because these individuals were disadvantaged when competing for food and habitat resources in the changing coastal environment, making them more vulnerable to starvation or predation. Further, in contrast to the Temperature-Size Rule, we demonstrate that only a portion of the large summer increase in body size during MHWs could be explained by elevated growth rates in the nursery. These results indicate that factors other than growth rate, likely size-selection, influenced the size of post-settlement Pacific cod during the MHWs, and these factors may become exacerbated as coastal habitats continue to change during future periods of warming. Sampling only the “super survivors” can mask negative impacts that may be occurring between sampling periods, highlighting a need to consider fine-scale in situ data of individuals across their life history when evaluating the responses of marine fishes to a changing climate.

## Methods

### Pacific cod early life history

GOA Pacific cod are highly fecund, single-batch spawners that produce demersal eggs in deep waters along the Alaskan continental shelf in early spring^77,78^. Upon hatching, larvae migrate to surface waters along the Alaska Peninsula and feed in the water column for 2–3 months, with pelagic juveniles (20–40 mm) observed as late as early June^79^. When they are between 30 and 40 mm long, juvenile Pacific cod settle into shallow (< 10 m deep), nearshore habitats along the Alaska Peninsula, with mid-June being the settlement period near Kodiak, AK^53^. During their first summer, juveniles primarily associate with submerged aquatic vegetation in shallow water less than 3 m below mean lower low water, although later in the summer, juveniles begin to school and associate with slightly deeper (4.5 m) less structured habitats^56^. In autumn, juveniles move to deeper (< 15 m) waters in coastal systems to overwinter and can remain in these nursery habitats as age-1 individuals^53,80^. As Pacific cod mature, they migrate offshore toward deeper and cooler continental shelf waters^81^.

### Temperature and marine heatwave classifications

Daily sea surface temperature data were collected between January 1983 through December 2019 from Alaska Department of Fish and Game temperature loggers located in Trident Bay, Kodiak, AK at ~ 10 m depth below mean lower low water^82^ (see Supplementary Fig. S7). This time series was selected due to its longevity, proximity to field collection sites, and ability to capture nearshore temperatures on Kodiak Island. Temperature data were processed using the R package *heatwaveR*^83^ to obtain MHW cumulative intensity values^84^ and MHW category classifications^85^. Additional sea surface temperature (°C) measurements taken with a YSI hand-held probe during field sampling in Anton Larsen Bay and Cook Bay were similar to temperatures recorded at Trident Bay. Based on MHW classifications for Trident Bay from 2006 to 2019, we binned years into three categories: ‘Before Heatwave’ (2006–2013); ‘Heatwave’ (2014–2016, 2019); and ‘Between Heatwave’ (2017–2018).

### Biological collections and abundance

Juvenile Pacific cod were collected from annual beach seine surveys on Kodiak Island, AK, USA between 2006 and 2019 as part of the NOAA Alaska Fisheries Science Center Fisheries Behavioral Ecology program (see Supplementary Fig. S7). This survey targets post-settlement age-0 Pacific cod in shallow structurally complex habitat consisting primarily of eelgrass (*Zostera marina*) and fleshy brown algae (*Saccharina* sp. and *Laminaria* sp.). Sampling was conducted twice per summer in mid-July and late August. Fish were collected from 8 sites in Anton Larsen Bay and 8 sites in Cook Bay along the northeastern coast of Kodiak Island during two successive days in July and August each year. Sample sizes were balanced so that sites were represented across all heatwave classes, and historically, these two bays have shown synchronized relationships in juvenile Pacific cod production^56^. Sites were 2–4 m below mean lower low water and were mostly sampled within 2 h of low tide^56^. Fish were captured using a demersal beach seine (36 m bag with 5-mm mesh; 1 m × 2.25 m wings with 13-mm mesh), sorted to species, counted, and measured (standard length, mm). Catch per unit effort (CPUE) was calculated as the number of age-0 Pacific cod per seine haul in July and August each year. Unlike Abookire et al.^46^, who used weighted models across a broad spatial scale in the GOA to assess patterns in juvenile Pacific cod abundance, we used raw CPUE on the Kodiak nursery grounds to examine relative abundance at the local scale. We used a linear mixed effects model to determine whether CPUE varied across months and heatwave classes, using year as a random effect.

Fish were frozen (− 20 °C) and returned to Hatfield Marine Science Center (HMSC) in Newport, OR for processing. No samples were available for July 2006, 2008, and 2011, or August 2011. Beach seine sampling was not conducted in August 2016. The biological data used in this study were collected as part of routine population monitoring to inform fisheries management. Field collections were completed independently by federal scientists at the NOAA Alaska Fisheries Science Center, who followed all internal federal policies and procedures as well as the American Fisheries Society policies on the Guidelines for Use of Fishes in Research (https://fisheries.org/docs/policy_useoffishes.pdf). Gulf of Alaska Pacific cod are considered a warm-adapted gadid, and juveniles were euthanized by rapid-chilling following capture in the field, with subsequent transfer to the freezer (− 20 °C) after ~ 3 h by federal scientists at NOAA. Collection permits were issued on an annual basis from the Alaska Department of Fish and Game to NOAA Alaska Fisheries Science Center staff and collaborators. Oregon State University’s Institutional Animal Care and Use Committee (IACUC) reviewed this study when analysis began in 2019 and provided an exemption for these collections because all samples were archival at the onset of this retrospective study. All methods are reported in accordance with ARRIVE guidelines^86^.

### Size and condition data

In the laboratory, samples were defrosted, weighed (to 0.01 mg) and measured (standard length, mm). Body condition indices were calculated using length–weight residuals and the hepatosomatic index (HSI). Length–weight condition residuals for all years were calculated using the log-linear model: ln(wet weight, g) = − 11.83 ± 0.096 + 3.07 ± 0.023 × ln(SL, mm) (R^2^_adj_. = 0.93; *P* < 0.0001; see Supplementary Fig. S8). HSI values were calculated as [liver wet weight (g)]/[whole body wet weight (g)] × 100. We used linear models for July and August to determine if yearly minimum standard length varied by heatwave class. We also used linear mixed effects models to determine whether standard length, body mass, length–weight condition residuals, or HSI values varied across months and heatwave classes, using year as a random effect. All available juveniles were used in statistical analyses comparing size and condition metrics across heatwave classes. For intra-annual analyses, we used juveniles only from years where samples had been collected in both July and August (see Supplementary Table S2 for more information on sample sizes).

### Diet composition analysis

Whole stomachs and total stomach contents were blotted dry and weighed to the nearest 0.01 mg. Prey items were analyzed under a dissecting microscope, quantified, and weighed to the nearest 0.001 mg. Stomach fullness was calculated as [weight (g) of stomach contents]/[total fish weight − weight of stomach contents]. Because there were empty stomachs in our analysis (n = 12), we square-root transformed our data to better allow for inclusion of those zero values in analysis. We used a linear mixed effects model to determine whether stomach fullness varied with month and heatwave class, using year as a random effect.

We evaluated July and August diet composition separately due to large differences in species composition observed between the two months. The relative importance of each prey group in the diet was quantified using the Prey-Specific Index of Relative Importance (PSIRI, Eq. 1), which provides a balanced treatment of the relative measures of prey quantity^87^. The PSIRI metric considers diet composition by the percent numerical abundance (%PN_i_) and percent weight (%PW_i_) of a prey item averaged over the number of stomach samples in which it occurs and the percent frequency of occurrence (%FO_i_), which is specific to each prey category. All PSIRI values from July and August prey groups were expressed as a percentage for each prey group in an individual stomach.

1 $${\%PSIRI}_{i }= \frac{{\%FO}_{i} \times ({\%PN}_{i}+ {\%PW}_{i})}{2}$$

For both months, a taxon was classified as a prey group if the PSIRI was greater than 3.5% of all stomachs sampled for that month across all years. Species that did not meet these criteria were grouped into an “Other” category for subsequent analysis. For a complete list of prey species, please see Supplementary Table S5. All abiotic items found in stomachs (e.g. rocks and plastic) and all empty stomachs were removed from subsequent diet composition analysis.

Non-metric Multidimensional Scaling (NMS) analysis was used to examine community diet relationships across MHW conditions. We used a Bray–Curtis distance measure and a random starting location with up to 200 iterations per run. Optimal dimensions for the solution were determined using scree plots. Stress, a goodness-of-fit criterion that measures the discrepancy between the distances of the original data set and the distances within the ordination space, was calculated for each ordination. Fit was further evaluated using Shepard plots, which plot the original dissimilarities of the data against the Bray–Curtis distances of the ordination^88^. The effect of individual points on each ordination was visualized to identify the proportion of overall variance explained by each individual stomach and confirm the robustness of the analysis (see Supplementary Fig. S9). Community diet data across MHW classes were visualized with dispersion ellipses of 95% confidence intervals of the average spatial scores. Species were overlaid as joint plots and correlated with the ordination axes using Pearson’s correlations to identify relationships. Standard length and water temperature were further visualized using contour plots overlaid onto the NMS ordination. All NMS analyses were run in the *vegan* package in R v. 4.2.2^89^ and visualized using the R package *ggplot2*^90^. By default, the metaMDS function in the *vegan* package penalizes the NMS for unequal ordination distance if ties are present and follows the ordination with a rotation of the principal axes to ensure that axis 1 reflects the principal source of variation^89^. We followed this with an additional rotation of the NMS to align with fish standard length for both months.

A nonparametric multi-response permutation procedure (MRPP) with a Bray–Curtis distance measure was used to determine if diets differed across MHW classes. A description of the effect size was provided by the chance-corrected within-group agreement statistic (*A*). We then used Indicator Species Analysis (ISA) to describe the primary prey species contributing to differences in diet composition between heatwave classes^91^. ISA is based on abundance and frequency of species between and within groups and uses indication values to determine faithfulness of a species to pre-determined groups. Statistical significance was determined with a Monte Carlo test. All ISA  analyses were run in the *indicspecies* package in R^92^.

To account for variability in fish size across heatwave classes, we examined the diet composition of a subset of individuals from July and August that had comparable sizes across the three heatwave classes. For July, we ran a second NMS ordination with sizes restricted to 50–60 mm (Before: n = 20; Heatwave: n = 32; Between: n = 17). For August, we ran an additional NMS ordination with sizes restricted to 70–90 mm (Before: n = 19; Heatwave: n = 17; Between: n = 13). We followed these size-restricted ordinations with MRPP and ISA analyses for each month.

### Otolith structural analysis

We used otolith structural analysis to generate growth estimates for fish collected in July and August. Otoliths are metabolically inert, calcium carbonate structures in the inner ear of teleost fishes that grow incrementally, laying down daily protein- and calcium carbonate-rich bands which can be used to determine growth of individual fish through time^93,94^. Otolith size and body size are highly correlated in Pacific cod^95,96^, and daily formation of otolith increments has been validated for Pacific cod at 10 °C up to 120 days post-hatch^97^.

We mounted left or right sagittae on glass slides using thermoplastic resin and polished to expose the core in the transverse plane using Wetordry paper (800–2000 grit), Buehler lapping film (3–30-micron grit), and alumina slurry (0.3 micron). Polished otoliths were imaged at 100 × and 400 × magnification using a Leica DM1000 compound microscope and a Levenhuk M1000 digital camera. Otolith radius and daily increments were counted and measured along the otolith’s proximal–distal axis using ImagePro Premier software to determine daily nursery growth within a 21-day period prior to sampling. 21 days was selected as our period of interest because it was likely to encompass nursery processes for both the July and August fish^53^. Each otolith was interpreted at least 2 times by one to three readers. If independent counts varied by > 10%, otoliths were revisited and discrepancies resolved. No otoliths were discarded from analysis due to inconsistencies in reading.

### Back-calculated size and growth

We estimated daily size of individuals based on changes in estimated daily size, which was back-calculated using daily otolith radii measurements. We then estimated relative growth (mm/mm/day) for 21 days prior to capture. To accomplish this, we first examined the relationship between the otolith radius and the fish’s size at capture in July and August for all years of the analysis. The relationship between standard length and otolith radius was linear, but annually variable (see Supplementary Fig. S10). Therefore, we used the Biological Intercept model^98^ (Eq. 2) to back-calculate daily sizes from otolith increment for each year of our analysis. We calculated the standard length of an individual at age *a* (*L*_*a*_) as:

2 $${L}_{a}= {L}_{c}+ \frac{\left({O}_{a}-{O}_{c}\right)\times \left({L}_{c}-{L}_{0}\right)}{\left({O}_{c}-{O}_{0}\right)}$$

where *L*_*c*_ is the length at capture, *O*_*a*_ is the otolith radius at age *a*, *O*_*c*_ is the otolith radius at capture, and *L*_0_ and *O*_0_ define the biological intercept of the length (*L*_0_ = 3.9 mm) and otolith radius (*O*_0_ = 8.3 µm), using published biological intercept values for Pacific cod from Narimatsu et al.^97^. After calculating the back-calculated size of an individual for each of the 21 days prior to capture, we determined daily relative growth rates (mm/mm/day) (see Supplementary Fig. S11). We used relative growth rates as opposed to absolute growth rates for subsequent analysis because fish sizes were highly variable across years and heatwave classes^99^.

### Nursery growth analysis

We compared nursery growth between July and August before, during, and between MHW events. We used model selection on linear mixed models to assess factors influencing nursery growth rate in July and August. Global models for July and August included the same fixed effects: standard length; surface ocean temperatures from Trident Bay; juvenile Pacific cod abundance from Kodiak beach seine surveys; Axis 1 scores from the NMS analysis (proxy for diet composition); stomach fullness; and heatwave class. We also included the fish ID as a random intercept in the global models because we repeatedly measured otolith increment widths from the same individuals, and we included otolith increment number from the final 21 days of life as a random slope because we sampled fish at different ages with different capture dates^100^.

We selected the optimal random, fixed, and error structure of the model using a three-step process^101^. First, we determined the optimal random effects structure by considering models with a random intercept of fish ID and a random slope for day of life. Next, we determined the most parsimonious fixed effects structure by considering all possible fixed effects from the global models. Finally, we determined the optimal error structure by considering a model that accounted for temporal autocorrelation with an auto-regressive model of order 1 (AR1). For each step, we compared the Akaike information criterion (AIC) score between models to determine best fit^102^. For a complete list of models tested, see Supplementary Table S6. All continuous predictor variables were scaled by dividing the centered values by their standard deviations to facilitate model convergence and interpretation of interaction terms, and we used a “Nelder-Mead” model optimizer to facilitate model convergence. Nursery growth was log-transformed to satisfy model assumptions. We validated best-fit models for July and August by examining plots of residuals vs. fitted values, residuals vs. predictor variables, and quantile–quantile (QQ) plots. All analyses were performed in R v. 4.2.2 using the *lme4*^103^ and *nlme*^104^ packages and visualized in *ggplot2*^90^.

We extracted marginal means from our final July and August growth models to examine relationships between predicted growth rate and specific terms and interactions of interest within the model. Marginal means are calculated by averaging specific model variables of interest over the values or levels of the remaining non-focal predictors in the model^105^. This allows for the interpretation of specific model terms when all other non-focal terms in the model are held constant. To see a full list of marginal means for both months, see Supplementary Table S7. Marginal means were extracted using the R packages *effects*^106^ and *ggeffects*^105^.

### August size predictions

We explored the potential for faster growth to account for the notable increase in body size between July and August that occurred only during MHWs. We predicted August sizes for July individuals based on their size at capture, average July growth rates, and the average number of days between July and August sampling. July growth rates were used instead of August growth rates because we were interested in growth patterns over an average of 40 days (rather than 21 days) between the July and August sampling periods; the August population represented only surviving individuals; and growth declined at larger sizes (Fig. 4) such that July growth rates would represent the upper estimates of growth during this period. We then compared these predicted August sizes to the true sizes of the individuals captured in August using a linear mixed effects model with heatwave as a fixed effect and year as a random effect. We also examined size quantiles from July to determine whether predicted August sizes could match actual August sizes if only certain size classes survived to August during MHWs.

We also compared our predicted and actual August sizes to six simulated populations of 10,000 individuals (one each for predicted August sizes and observed August sizes across the three MHW classes) to further evaluate potential model uncertainty associated with the predicted length distributions. We randomly drew samples from this simulated population such that sample sizes were equivalent to our observed sample sizes in the field (Table 1). Finally, we examined different size quantiles of our simulated population to assess whether the predicted August size distributions could match the actual simulated August distributions if only certain size quantiles were used.

### Supplementary Information

Supplementary Information.

## Data Availability

Data and code are available in a GitHub repository: https://github.com/hthalmann/Thalmann_et_al_MHWs_alter_nursery_habitat_for_GOA_Pacific_Cod.
