# Supplementary material for: Marine heatwaves alter the nursery function of coastal habitats for juvenile Gulf of Alaska Pacific cod
Source: Sci Rep. 2024 Jun 27;14:14018. doi: 10.1038/s41598-024-63897-w (PMC11211443; doi:10.1038/s41598-024-63897-w)
Supplement: Supplementary file 1 — Supplementary Information. [file 41598_2024_63897_MOESM1_ESM.pdf]

Supplemental Material for:

Marine Heatwaves alter the nursery function of coastal habitats for juvenile Gulf of Alaska Pacific cod

Hillary L. Thalmann[1,*], Benjamin J. Laurel[2], L. Zoe Almeida[1], Kaitlyn E. Osborne[1], Kaylee Marshall[1], Jessica A. Miller[1]

[1]Oregon State University Department of Fisheries, Wildlife, and Conservation Sciences; Coastal Oregon Marine Experiment Station; Hatfield Marine Science Center, 2030 SE Marine Science Dr., Newport, OR 97365

[2]NOAA Alaska Fisheries Science Center, Hatfield Marine Science Center, 2030 SE Marine Science Dr., Newport, OR 97365

*Corresponding author: hillary.thalmann@oregonstate.edu

ORCIDs:

HLT: 0000-0002-2112-5131. BJL: 0000-0001-7150-0879. LZA: 0000-0003-0280-5964. KEO: 0009-0003-0880-7984. JAM: 0000-0002-6491-4085

Table S1: Marine heatwave classifications for Trident Bay, Kodiak Island, AK from January 2005 to December 2019, including event category (based on categorization from Hobday et al. 2018); season of the event, duration of the event (including start date, end date, and peak date), and event intensity cumulative, mean, and maximum). All long-term data was collected through the Alaska Department of Fish and Game. Between 1 Jan 2014 and 31 Dec 2016, heatwave conditions occurred in 748 of 1096 days and consisted of 12 discrete events. The longest of these events was 373 days between 26 Nov 2015 and 2 Dec 2016, with the highest positive temperature anomaly (peak intensity) of 3.07ºC occurring on 20 July 2016. In 2019, heatwave conditions occurred in 332 of 365 total days with a peak intensity of 3.51ºC occurring on 16 Aug 2019.

Event	Category	Season	Duration (d)	Start Date	Peak Date	End Date	Cumulative Intensity (I_{cum})	Mean Intensity	Maximum Intensity
1	I Moderate	Winter	9	1/2/06	1/5/06	1/10/06	11.06	1.23	1.28
2	I Moderate	Summer/Fall	34	9/4/10	9/20/10	10/7/10	35.05	1.03	1.33
3	I Moderate	Winter	18	1/23/14	2/2/14	2/9/14	28.34	1.57	1.77
4	I Moderate	Spring	5	6/12/14	6/15/14	6/16/14	4.88	0.98	1.02
5	I Moderate	Spring/Summer	13	6/29/14	7/8/14	7/11/14	14.87	1.14	1.44
6	I Moderate	Summer	11	8/4/14	8/8/14	8/14/14	16.00	1.45	1.88
7	I Moderate	Summer	9	9/21/14	9/26/14	9/29/14	8.07	0.90	0.94
8	II Strong	Fall/Winter	92	11/7/14	1/19/15	2/6/15	171.24	1.86	2.38
9	I Moderate	Winter	29	2/11/15	3/7/15	3/11/15	55.31	1.91	2.23
10	I Moderate	Winter/Spring	25	3/19/15	3/27/15	4/12/15	43.30	1.73	1.94
11	II Strong	Spring/Summer	47	5/31/15	6/28/15	7/16/15	80.17	1.71	2.27
12	II Strong	Summer/Fall	113	7/23/15	8/27/15	11/12/15	176.25	1.56	2.48
13	III Severe	Year-round	373	11/26/15	7/20/16	12/2/16	784.86	2.10	3.07
14	I Moderate	Fall	13	12/9/16	12/18/16	12/21/16	15.95	1.23	1.46
15	I Moderate	Spring	8	6/20/17	6/26/17	6/27/17	8.47	1.06	1.15
16	I Moderate	Fall/Winter	35	12/7/17	12/17/17	1/10/18	44.79	1.28	1.54
17	I Moderate	Winter	5	1/16/18	1/18/18	1/20/18	7.03	1.41	1.46
18	II Strong	Summer/Fall	100	9/8/18	9/20/18	12/16/18	147.94	1.48	2.15
19	I Moderate	Winter	21	1/24/19	2/11/19	2/13/19	29.50	1.40	1.55
20	III Severe	Year-round	311	2/18/19	8/16/19	12/25/19	589.89	1.90	3.51

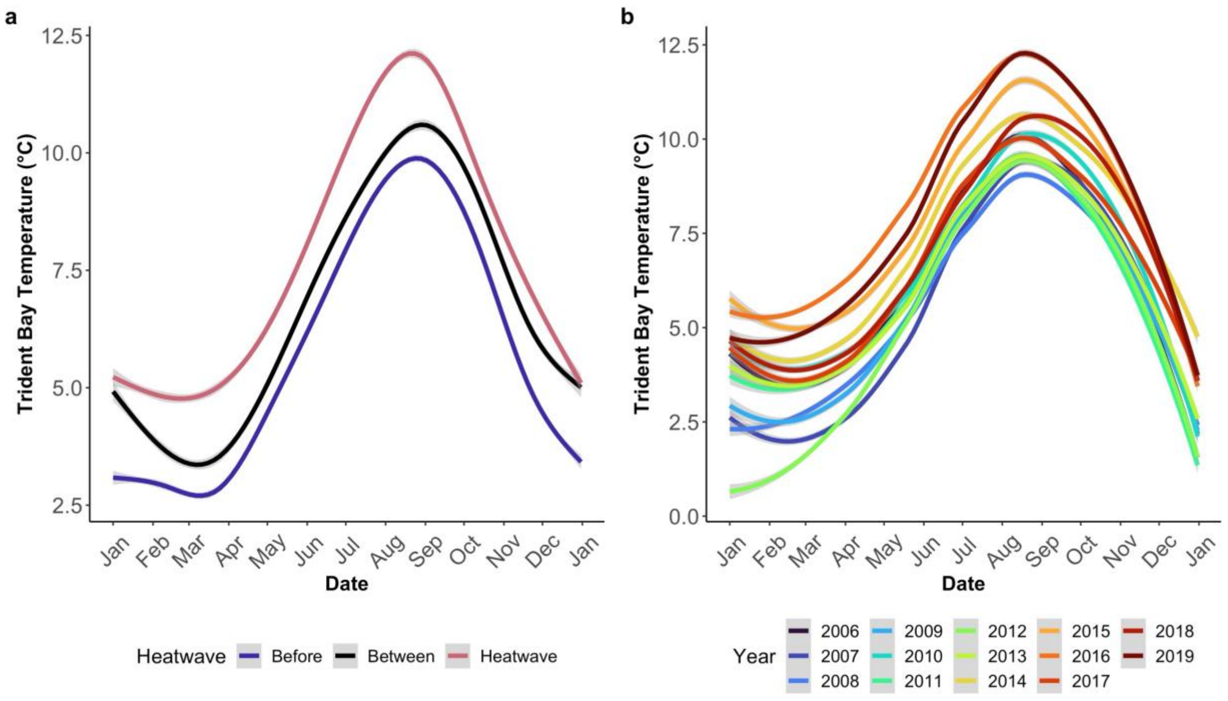

Figure S1: Water temperatures from Trident Bay, Kodiak, AK were >3°C warmer during marine heatwave events than temperatures prior to the heatwaves. Water temperatures between the heatwaves (2017-2018) remained higher than normal, although not as warm as the heatwaves. Temperature throughout the year is shown by heatwave class (left) and by year (right) from 2006 to 2019. Shaded polygons represent 95% confidence intervals. Water samples are collected from 10m below mean lower low water.

Table S2: Sample sizes for total catch, diet composition, and growth analyses of juvenile Pacific cod by year for July and August sampling. Summary statistics are reported for Trident Bay, AK temperature (ºC), age-0 Pacific cod catch per unit effort (CPUE), standard length (mm), and body mass (g). Samples were not available for July 2006, 2008, and 2011, or August 2011 and 2016. Summary statistics are reported as mean ± standard error.

Sampling Month	Year	Heatwave Class	Total *n*	Diet *n*	Growth *n*	Trident Bay Mean Temp. (°C)	CPUE	Standard Length (mm)	Body Mass (g)
July	2006	Before	--	--	--	9.09 ± 0.08	122.55 ± 35.92	--	--
	2007	Before	31	23	28	8.58 ± 0.12	8.84 ± 3.34	43.9 ± 0.5	0.53 ± 0.02
	2008	Before	--	--	--	7.92 ± 0.08	59.33 ± 29.09	--	--
	2009	Before	68	27	33	8.99 ± 0.08	6.06 ± 3.38	39.2 ± 0.7	0.67 ± 0.07
	2010	Before	104	25	23	8.69 ± 0.09	19.25 ± 10.48	45.2 ± 0.5	1.08 ± 0.04
	2011	Before	--	--	--	8.87 ±0.12	29.39 ± 10.90	--	--
	2012	Before	71	25	34	8.77 ± 0.05	285.71 ± 62.19	41.1 ± 0.7	0.73 ± 0.04
	2013	Before	41	25	19	9.19 ± 0.19	12.16 ± 5.71	49.4 ± 0.8	1.08 ± 0.06
	2014	Heatwave	39	26	20	9.92 ± 0.09	10.94 ± 5.40	50.7 ± 1.2	1.27 ± 0.0.8
	2015	Heatwave	9	9	9	10.40 ± 0.08	0.15 ± 0.09	47.7 ± 1.3	1.01 ± 0.11
	2016	Heatwave	49	25	20	11.70 ± 0.11	2.50 ± 1.35	63.5 ± 0.9	2.68 ± 0.13
	2017	Between	32	26	24	9.31 ± 0.07	166.19 ± 30.03	59.9 ± 2.0	2.41 ± 0.24
	2018	Between	46	25	20	9.53 ± 0.08	99.69 ± 31.54	64.6 ± 2.0	3.15 ± 0.32
	2019	Heatwave	31	24	20	11.60 ± 0.07	5.25 ± 3.01	49.4 ± 1.2	1.47 ± 0.12
August	2006	Before	285	27	23	10.40 ± 0.03	77.88 ± 12.84	67.9 ± 0.6	3.57 ± 0.12
	2007	Before	38	21	25	9.50 ± 0.07	3.13 ± 1.26	53.9 ± 1.1	0.95 ± 0.06
	2008	Before	175	26	20	9.50 ± 0.05	9.56 ± 2.05	67.9 ± 0.8	2.20 ± 0.09
	2009	Before	135	26	25	9.14 ± 0.06	58.75 ± 40.10	66.2 ± 0.8	3.32 ± 0.11
	2010	Before	12	12	12	10.03 ± 0.09	2.03 ± 0.93	70.5 ± 2.8	4.37± 0.55
	2011	Before	--	--	--	10.06 ± 0.04	27.67 ± 10.60	--	--
	2012	Before	93	25	19	9.26 ± 0.06	33.96 ± 8.50	65.7 ± 0.9	3.11 ± 0.17
	2013	Before	39	25	20	9.79 ± 0.09	7.56 ± 2.00	76.3 ± 1.7	4.75 ± 0.38
	2014	Heatwave	3	3	3	11.05 ± 0.08	1.75 ± 0.75	103.0 ± 4.7	11.44 ± 1.59
	2015	Heatwave	24	24	22	11.97 ±0.09	3.81 ± 3.61	93.9 ± 1.5	9.16 ± 0.50
	2016	Heatwave	--	--	--	12.10 ± 0.07	--	--	--
	2017	Between	30	25	25	10.30 ± 0.06	35.22 ± 10.75	80.2 ± 2.1	5.37 ± 0.38
	2018	Between	36	25	19	10.21 ± 0.06	59.69 ± 24.91	87.1 ± 3.6	8.51 ± 1.05
	2019	Heatwave	29	26	25	13.13 ± 0.08	1.41 ± 0.96	90.6 ± 2.4	9.80 ± 1.10

Table S3: Type III SS ANOVA results for evaluating the impact of heatwave (before, during, between) and month (July and August) on:1) Abundance; 2) Standard Length; 3) Body Mass; 4) HSI Condition; 5) HSI condition without 2019, and 6) Length-Weight Condition Residuals.

Predictor	Chi-Square	df	*P*-value
**Abundance**			
Number of obs: n = 27; Groups: Year = 14			
Intercept	3.5	1	0.060
Heatwave	1.2	2	0.555
Month	1.5	1	0.222
Heatwave x Month	0.6	2	0.757
**Standard Length**			
Number of obs: n = 1,420; Groups: Year = 13			
Intercept	11507.4	1	< 0.0001
Heatwave	28.1	2	< 0.0001
Month	1196.7	1	< 0.0001
Heatwave x Month	74.0	2	< 0.0001
**Body Mass**			
Number of obs: n = 1,420; Groups: Year = 13			
Intercept	7.2	1	0.007
Heatwave	19.6	2	< 0.0001
Month	1090.6	1	< 0.0001
Heatwave x Month	73.3	2	< 0.0001
**HSI Condition**			
Number of obs: n = 1,346; Groups: Year = 13			
Intercept	0.44	1	0.507
Heatwave	11.4	2	0.003
Month	15.3	1	< 0.0001
Heatwave x Month	69.1	2	< 0.0001
**HSI Condition (No 2019)**			
Number of obs: n = 1,286; Groups: Year = 12			
Intercept	1.51	1	0.219
Heatwave	6.76	2	0.034
Month	19.9	1	< 0.0001
Heatwave x Month	3.56	2	0.169
**LW Condition Residuals**			
Number of obs: n = 1,420; Groups: Year = 13			
Intercept	0.85	1	0.357
Heatwave	1.29	2	0.524
Month	2.44	1	0.118
Heatwave x Month	2.42	2	0.299

Stomach Fullness			
Number of obs: n = 525; Groups: Year = 13			
Intercept	143.4	1	< 0.0001
Heatwave	0.42	2	0.812
Month	14.0	1	0.0002
Heatwave x Month	9.46	2	0.0088

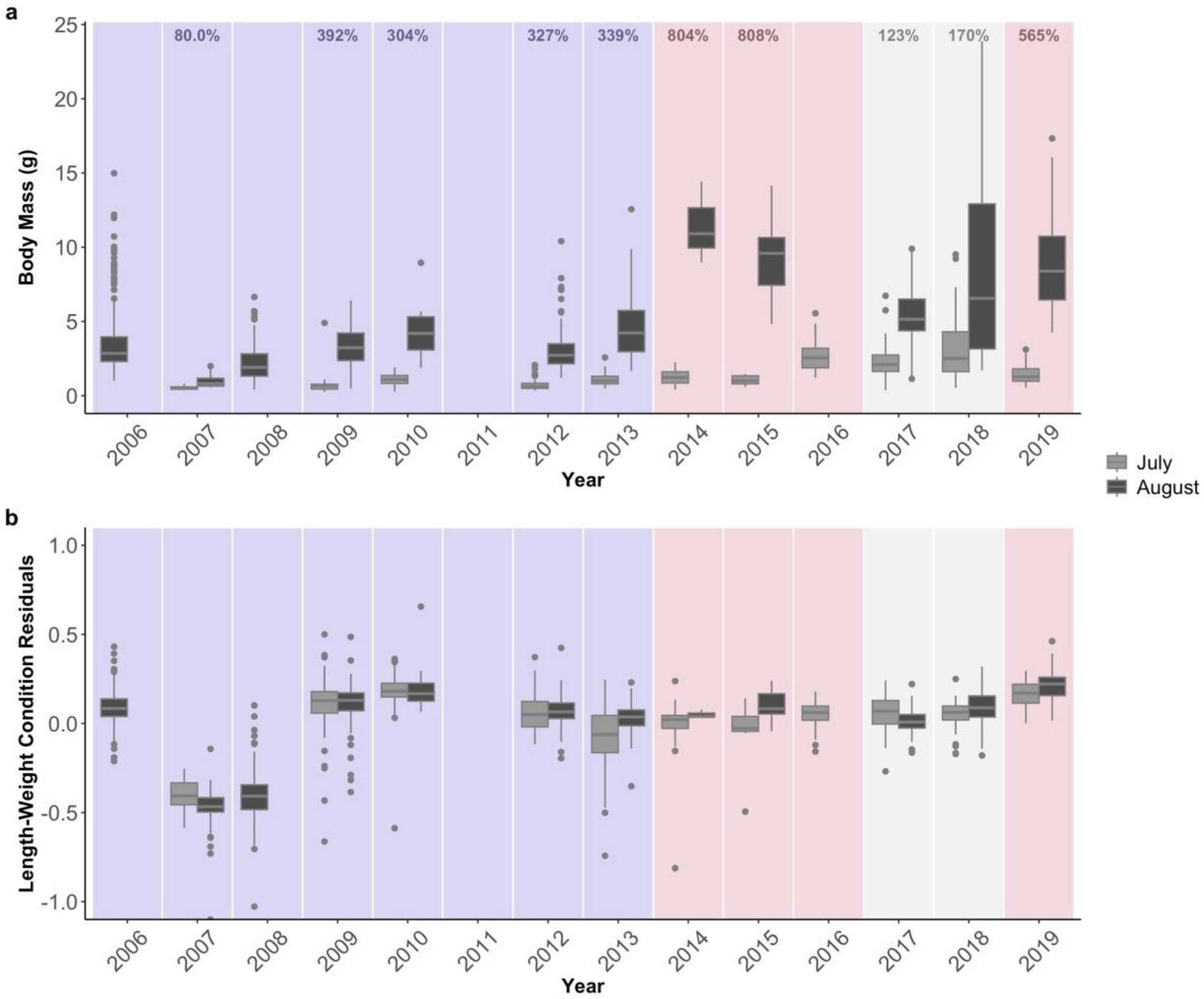

Figure S2: Juvenile Pacific cod body mass (a) and length-weight condition residuals (b) in July (light grey) and August (dark grey) from 2006-2019. Plots are shaded based on their heatwave class, with blue representing years before the MHWs, red representing years during MHWs, and light grey representing years between MHWs. Boxplots extend from the first to the third quartiles of the data, with whiskers that extend to the largest values no further than the 1.5*IQR. Individually plotted points represent outliers beyond the 1.5*IQR range.

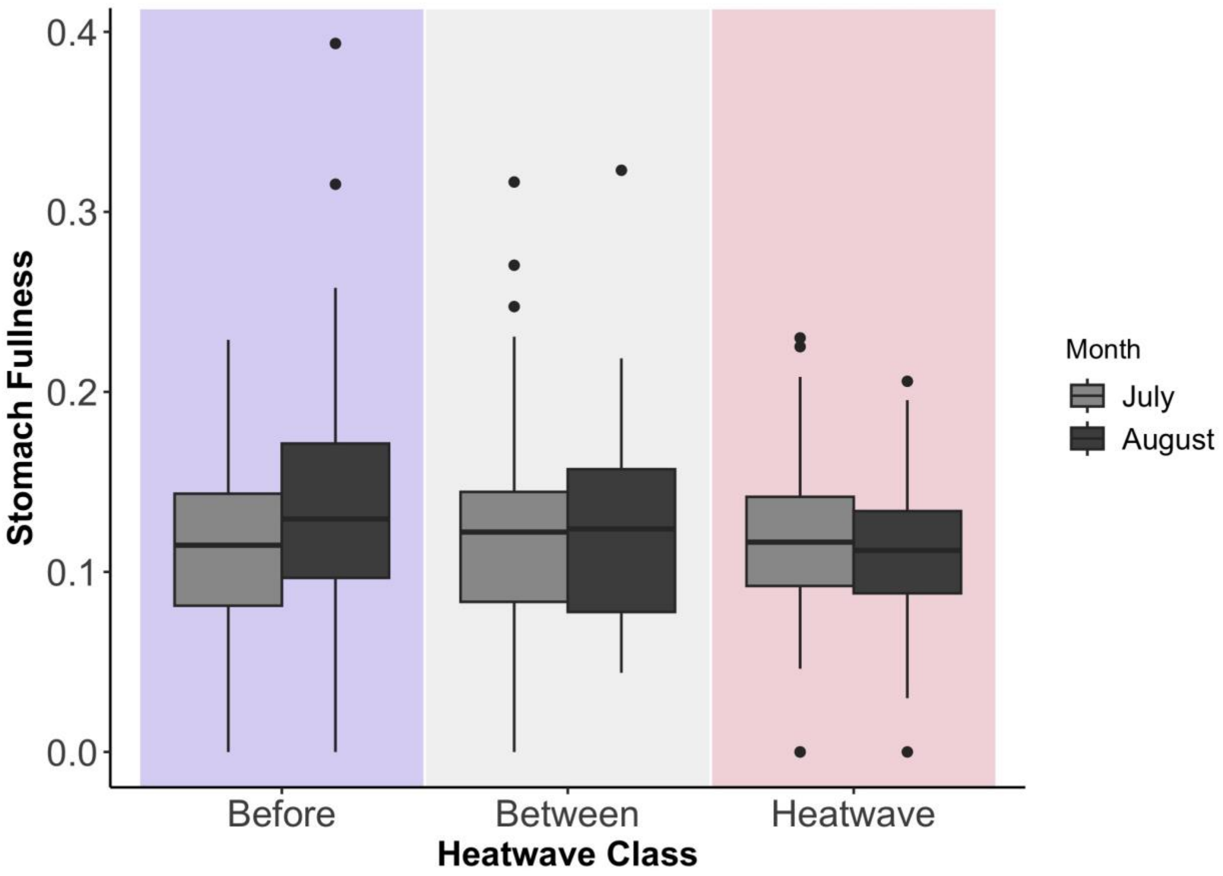

Figure S3: Juvenile Pacific cod square root-transformed stomach fullness in July and August did not vary across marine heatwave events. Light-grey boxplots represent July stomach fullness, and dark-grey boxplots represent August stomach fullness. Plots are shaded based on their heatwave class, with blue representing years before the MHWs, red representing years during MHWs, and grey representing years between MHWs. Boxplots extend from the first to the third quartiles of the data, with whiskers that extend to the largest values no further than the 1.5*IQR. Individually plotted points represent outliers beyond the 1.5*IQR range.

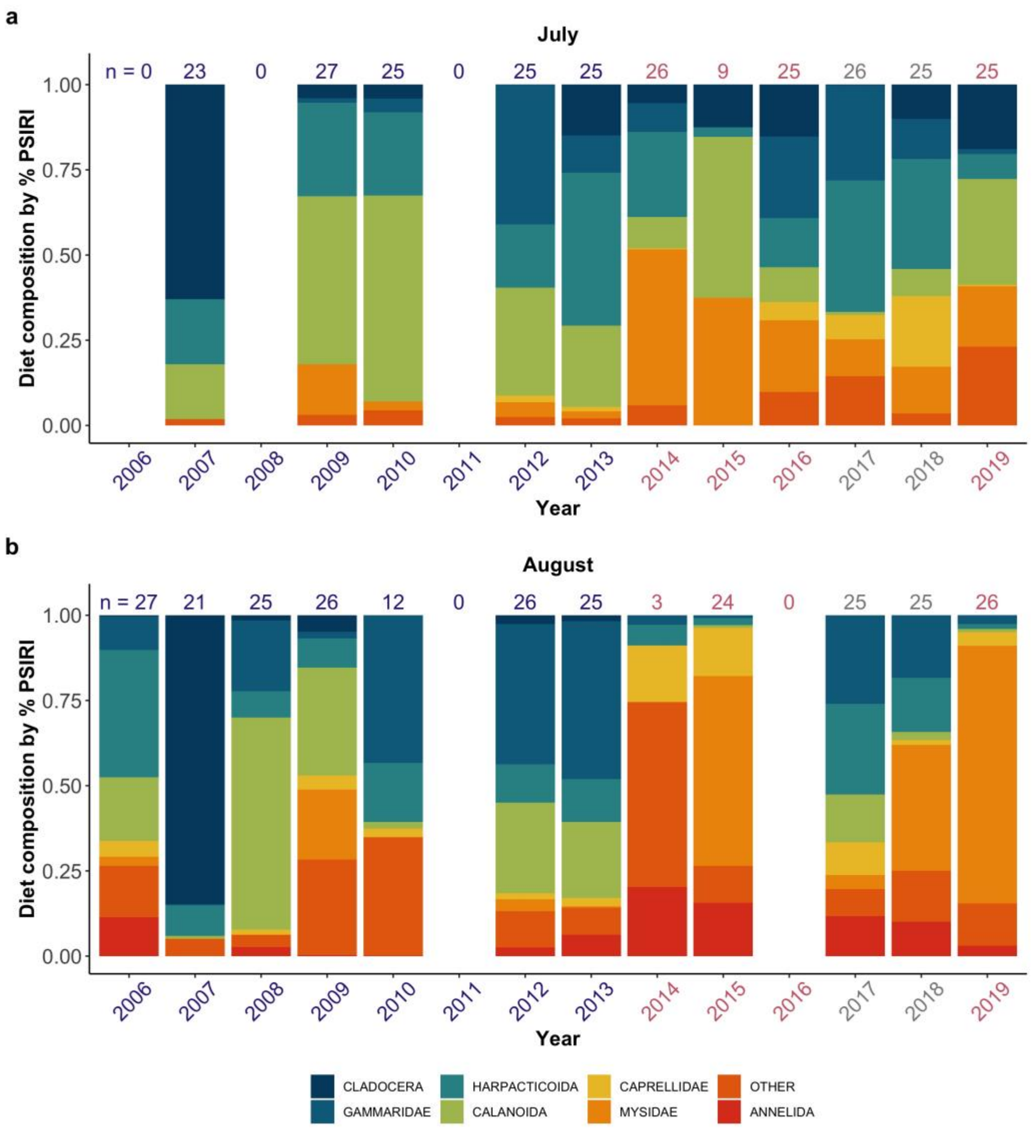

Figure S4: Juvenile Pacific cod diet composition (represented by year) varied by year and by heatwave class, with mysids representing a large portion of the diet by August in marine heatwave years. Diet composition is represented by percent Prey Specific Index of Relative Importance (PSIRI) between 2006 and 2019 for July (top panel) and August (lower panel). The "other prey" category includes all prey that was not found in at least 3.5% of all stomachs sampled. Years are colored by their heatwave class, with blue representing years before marine heatwaves, red representing years during marine heatwaves, and grey representing years between marine heatwaves.

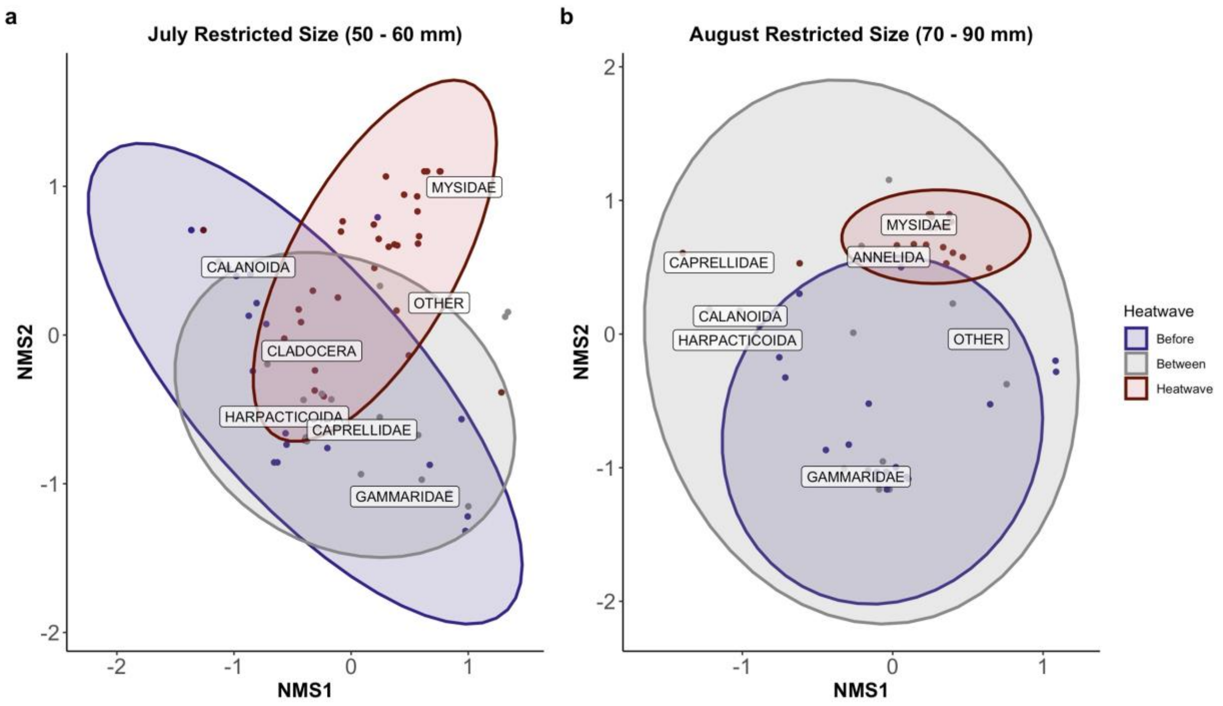

Figure S5: Size-restricted nonmetric multidimensional scaling (NMS) biplot of July (right) and August (left) diet composition for juvenile Pacific cod from 2007-2019 in heatwave conditions (red), before heatwaves (blue), and between heatwaves (grey). Sizes were restricted to 50-60 mm standard length in July and 70-90 mm standard length in August. Ellipses represent 95% confidence intervals in the two major axes.

Table S4: Type III SS ANOVA results for evaluating the impact of heatwave (before, during, between) and grouping variable (actual August size and predicted August size) on August standard length.

Predictor	Chi-Square	df	*P*-value
**Actual and Predicted August Growth**			
Number of obs: n = 669; Groups: Year = 10			
Intercept	879.6	1	< 0.0001
Heatwave	50.71	2	< 0.0001
Grouping Factor (Actual Size or Predicted Size)	0.08	1	0.7713
Heatwave x Group	82.5	2	< 0.0001

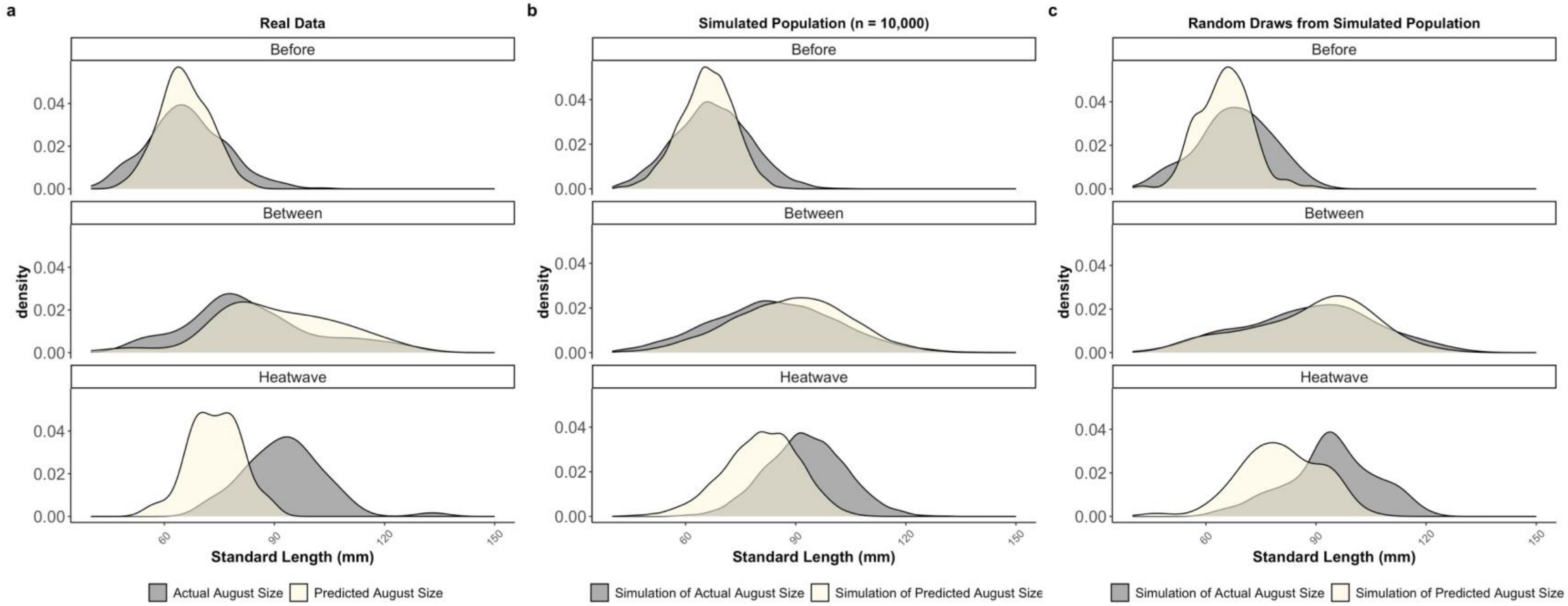

Figure S6: Predicted August size distributions (beige) and actual August size distributions (grey) for (a) real data collected from the field; (b) six simulated populations of 10,000 individuals (one each for predicted August sizes and observed August sizes across the three MHW classes); and (c) random draws of smaller subsets of the large, simulated population. Predicted August sizes were based on July sizes and July growth rates and calculated by summing mean July growth rates for that year over the number of days between July and August sampling in that year.

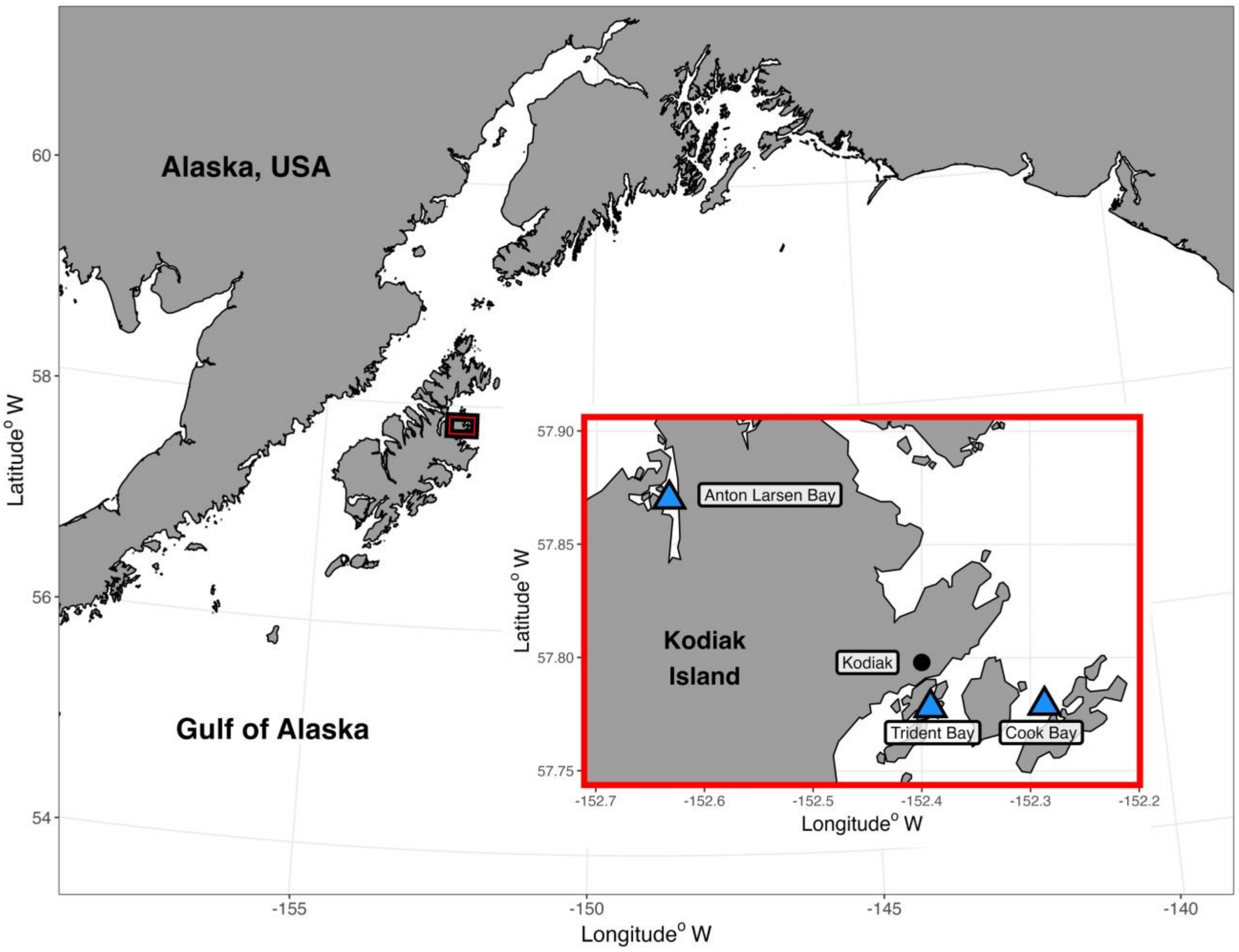

Figure S7: Juvenile Pacific cod were sampled via beach seine surveys from Anton Larsen Bay and Cook Bay on the northeastern coast of Kodiak Island, Alaska, U.S.A. in July and August 2006-2019. Daily nearshore temperature readings were taken from long-term temperature loggers in Trident Bay on the northeastern coast of Kodiak Island.

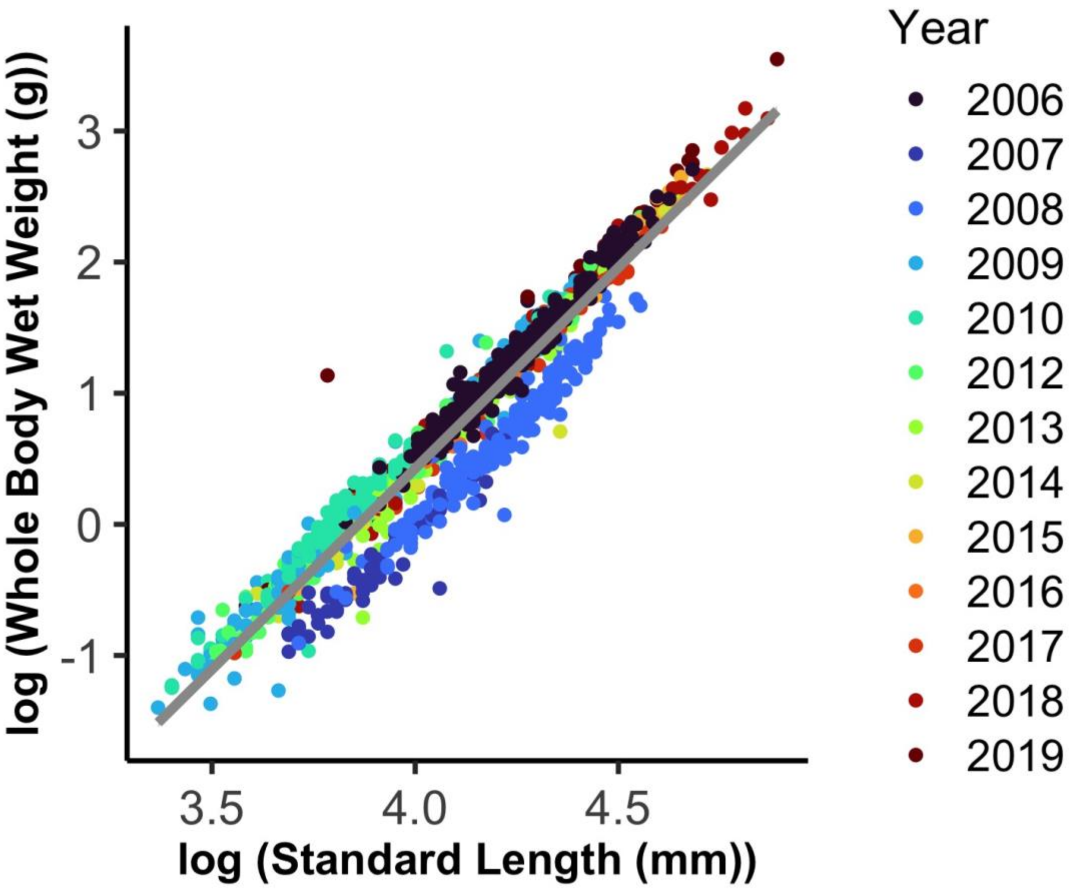

Figure S8: Relationship between juvenile Pacific cod log-transformed whole body wet weight (g) and log-transformed standard length (mm) from both July and August between 2006 and 2019.

Table S5: July and August prey lists.

July Diet Composition								
**Before MHWs**			**Between MHWs**			**During MHWs**		
**NMS Prey Group**	**Species**	**% PSIRI**	**NMS Prey Group**	**Species**	**% PSIRI**	**NMS Prey Group**	**Species**	**% PSIRI**
**Small Calanoid Copepods**		**36.5%**	**Small Calanoid Copepods**		**4.3%**	**Small Calanoid Copepods**		**19.8%**
	Acartia longiremis			*Acartia longiremis*			*Acartia longiremis*	
	Acartia spp.			*Acartia* spp.			*Acartia* spp.	
	Metridia spp.			*Metridia* spp.			*Calanus marshallae*	
	Calanus spp.			*Calanus* spp.			*Calanus* spp.	
	Unidentified Small Calanoid Copepod			Unidentified Small Calanoid Copepod			*Metridia* spp.	
							Eurytemora spp.	
							Unidentified Small Calanoid Copepod	
**Harpacticoid Copepods**		**27.1%**	**Harpacticoid Copepods**		**35.5%**	**Harpacticoid Copepods**		**14.3%**
	Diosaccus spinatus			*Diosaccus spinatus*			*Diosaccus spinatus*	
	Orthopsyllus linearis			*Orthopsyllus linearis*			*Orthopsyllus linearis*	
	Tisbidae			Tisbidae			Tisbidae	
	Unidentified Harpacticoid			Unidentified Harpacticoid			Unidentified Harpacticoid	
**Cladocerans**		**16.2%**	**Cladocerans**		**5.0%**	**Cladocerans**		**13.1%**
	Podon spp.			*Podon* spp.			*Podon* spp.	
**Caprellid Amphipods**		**0.7%**	**Caprellid Amphipods**		**13.9%**	**Caprellid Amphipods**		**1.8%**
	Caprillidea			Caprillidea			Caprillidea	
**Gammarid Amphipods**		**12.0%**	**Gammarid Amphipods**		**20.0%**	**Gammarid Amphipods**		**10.2%**
	Unidentified Gammarid			Corphidae			Unidentified Gammarid	
				Unidentified Gammarid				
**Mysids**		**4.7%**	**Mysids**		**12.2%**	**Mysids**		**29.1%**
	Exacanthomysis arctopacifica			*Exacanthomysis arctopacifica*			*Exacanthomysis arctopacifica*	
	Neomysis kadiakensis			*Neomysis kadiakensis*			*Neomysis kadiakensis*	
	Mysis spp.			*Mysis* spp.			*Mysis* spp.	
	Unidentified Mysid			Unidentified Mysid			Unidentified Mysid	
**Other**		**2.8%**	**Other**		**9.1%**	**Other**		**11.7%**
	Clam (*Megayoldia* spp.)			Unidentified fish larvae			Clam (*Megayoldia* spp.)	
	Moon Snail (*Neverita* spp.)			Moon Snail (*Neverita* spp.)			Moon Snail (*Neverita* spp.)	
	Cumacea			Insecta			Hyperiid Amphipod	
	Unidentified fish larvae			Annelid Worm (*Nereis* spp.)			Annelid Worm (*Nereis* spp.)	
	Hyperiid Amphipod			Cumacea			Hippolytid Shrimp	
	Cyclopoid Copepods (*Oithona* spp.)			Hyperiid Amphipod			Pteropoda	
	Unidentified crab zoea			Barnacle nauplii			Euphausiid (*Thysanoessa spinifera*)	
	Barnacle nauplii			Barnacle cyprids			Annelid Worm (*Nephtys* spp.)	
	Barnacle cyprids						Unidentified crab zoea	
							Unidentified Shrimp Larvae	
							Unidentified decapod	
							Barnacle nauplii	
							Barnacle cyprids	

August Diet Composition								
**Before MHWs**			**Between MHWs**			**During MHWs**		
**NMS Prey Group**	**Species**	**% PSIRI**	**NMS Prey Group**	**Species**	**% PSIRI**	**NMS Prey Group**	**Species**	**% PSIRI**
**Small Calanoid Copepods**		**25.9%**	**Small Calanoid Copepods**		**8.0%**	**Small Calanoid Copepods**		**0.9%**
	Acartia longiremis			*Acartia longiremis*			*Calanus marshallae*	
	Acartia spp.			*Acartia* spp.			*Calanus* spp.	
	Metridia spp.			*Metridia* spp.			*Eurytemora* spp.	
				Unidentified Small Calanoid Copepod				
	Calanus spp.							
	Eurytemora spp.							
	Unidentified Small Calanoid Copepod							
**Harpacticoid Copepods**		**15.0%**	**Harpacticoid Copepods**		**21.0%**	**Harpacticoid Copepods**		**1.9%**
	Diosaccus spinatus			*Diosaccus spinatus*			*Diosaccus spinatus*	
	Orthopsyllus linearis			*Orthopsyllus linearis*			*Orthopsyllus linearis*	
	Tisbidae			Tisbidae			Tisbidae	
	Unidentified Harpacticoid			Unidentified Harpacticoid			Unidentified Harpacticoid	
**Cladocerans**		**12.9%**	**Cladocerans**		**0.0%**	**Cladocerans**		**0.0%**
	Podon spp.			*Podon* spp.				
**Caprellid Amphipods**		**2.5%**	**Caprellid Amphipods**		**5.4%**	**Caprellid Amphipods**		**9.3%**
	Caprillidea			Caprillidea			Caprillidea	
**Gammarid Amphipods**		**22.0%**	**Gammarid Amphipods**		**22.0%**	**Gammarid Amphipods**		**1.8%**
	Ampithoidae			Corphidae			Unidentified Gammarid	
				Unidentified Gammarid				
	Corphidae							
	Unidentified Gammarid							
**Mysids**		**4.3%**	**Mysids**		**21.2%**	**Mysids**		**62.4%**
	Exacanthomysis arctopacifica			*Exacanthomysis arctopacifica*			*Exacanthomysis arctopacifica*	
	Neomysis kadiakensis			*Neomysis kadiakensis*			*Neomysis kadiakensis*	
	Mysis spp.			*Mysis* spp.			Unidentified Mysid	
	Unidentified Mysid			Unidentified Mysid				
**Annelid Worms**		**3.9%**	**Annelid Worms**		**10.8%**	**Annelid Worms**		**9.6%**
	Nereis spp.			*Nereis* spp.			*Nereis* spp.	
	Nephtys spp.			*Nephtys* spp.			Sipunculan	
	Sipunculan			Sipunculan				
**Other**		**13.5%**	**Other**		**11.6%**	**Other**		**14.1%**
	Clam (*Megayoldia* spp.)			Clam (*Megayoldia* spp.)			Clam (*Megayoldia* spp.)	
	Moon Snail (*Neverita* spp.)			Moon Snail (*Neverita* spp.)			Moon Snail (*Neverita* spp.)	
	Pteropoda			*Littorina* spp.			*Littorina* spp.	
	Salps			Pteropoda			Pteropoda	
	Cyclopoid Copepods (Oithona)			Hyperiid Amphipod			Barnacle Cyprid	
	Barnacle Nauplii			Isopoda			Hyperiid Amphipod	
	Barnacle Cyprid			Unidentified fish larvae			Isopoda	
	Cumacea			Cumacea			Cumacea	
	Hyperiid Amphipod			Insecta			Unidentified fish larvae	
	Isopoda			Hippolytid Shrimp			Hippolytid Shrimp	
	Isopod cryptoniscus			Pandalid Shrimp			Pandalid Shrimp	
	Fish Larvae (*Microgadus proximus*)			Unidentified decapod			Crangonid Shrimp	

Insecta<br>Hippolytid Shrimp<br>Cragonid Shrimp<br>Unidentified shrimp larvae<br>Unidentified decapod zoea<br>Unidentified decapod megalope	Crangonid Shrimp	Juvenile Fish (*Clupea pallasii*)

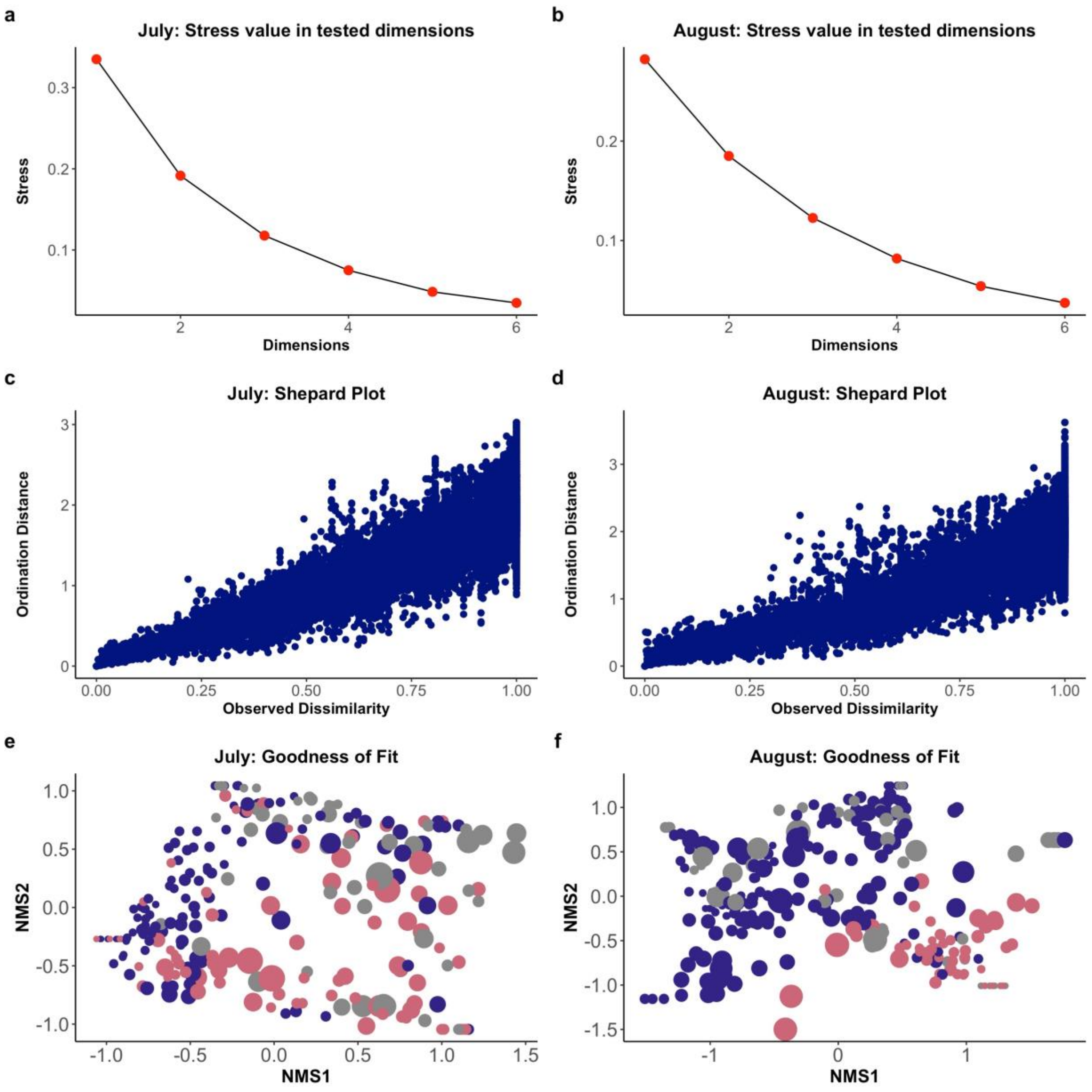

Figure S9: Top panel: scree plots for July (a) and August (b) assessing optimal number of dimensions for Non-Metric Multidimensional Scaling ordination based on overall stress. Middle panel: Shepard plots for July (c) and August (d) evaluating ordination fit. The observed dissimilarities within the data are plotted against the Bray-Curtis distances of the ordination. Bottom panel: goodness of fit for July (e) and August (f) to determine the proportion of overall variance explained by each point. Larger circles represent individual Pacific cod stomachs that account for a larger proportion of the overall variance within the ordination.

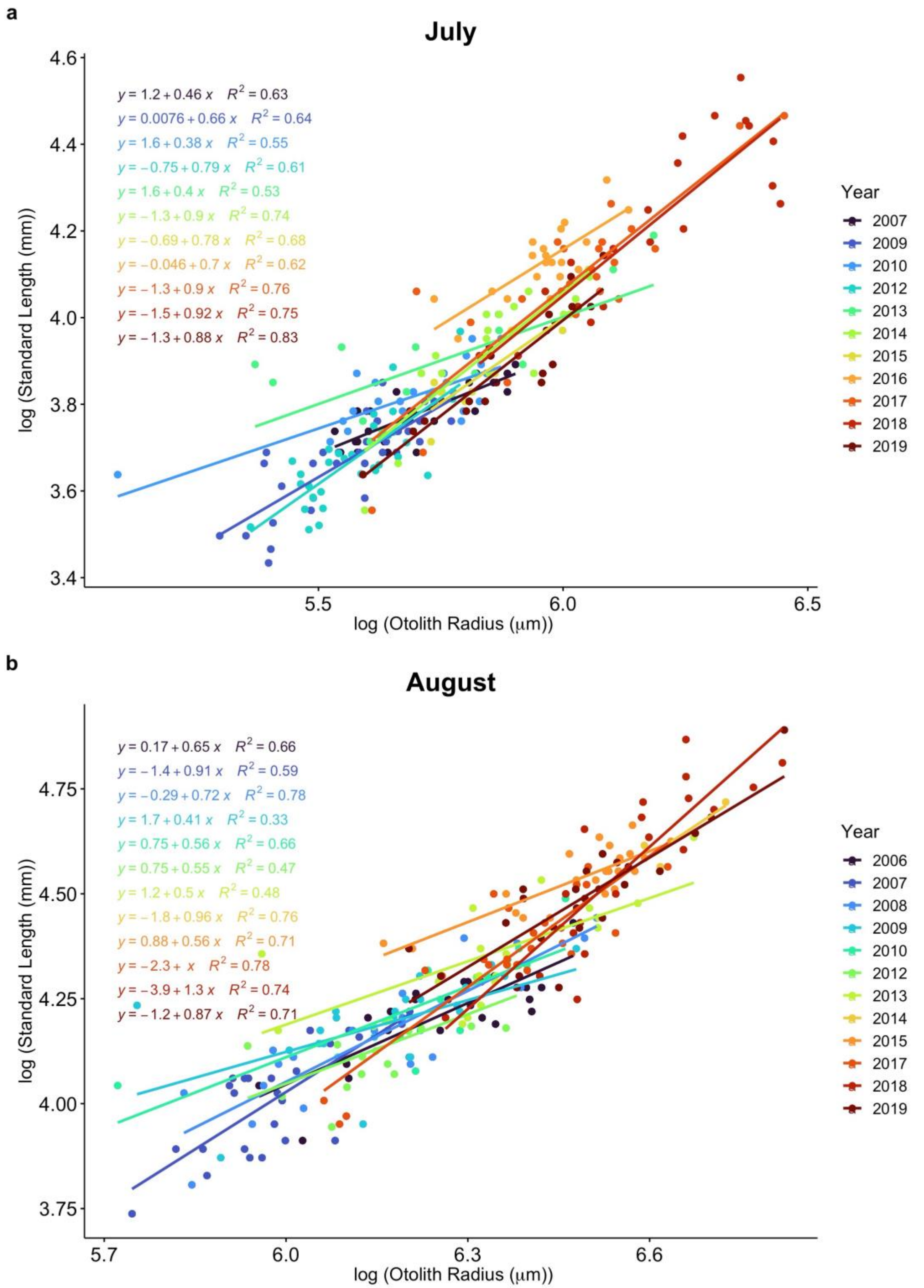

Figure S10: Relationship between juvenile Pacific cod log-transformed size (standard length, mm) and log-transformed otolith radius (μm) by year (2006-2019) for July (top) and August (bottom).

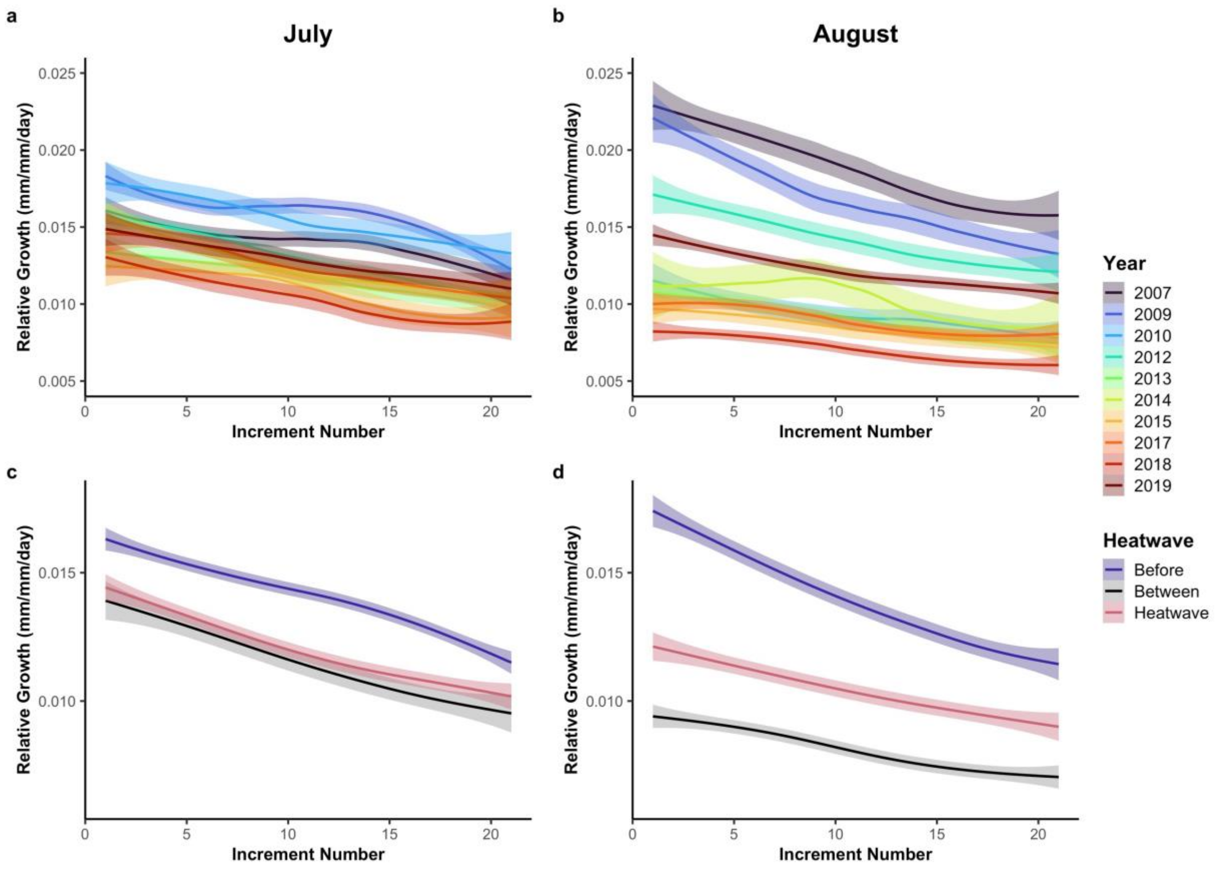

Figure S11: Mean daily relative growth trajectories (mm/mm/day); back-calculated from daily otolith increment widths using a Biological Intercept for the final three weeks of nearshore nursery residence by year (top) and heatwave class (bottom) for July (left) and August (right). Colored polygons represent 95% confidence intervals. Increment number 0 represents growth 21 days prior to capture. Increment number 21 represents growth on the final day prior to capture. Only years with data for both months are shown here.

Table S6: Steps for growth model selection, using a three-step process: 1: Determine optimal random structure; 2: Determine optimal fixed structure; 3: Determine optimal error structure. All predictor variables were scaled and centered.

July		August	
**Step 1: Optimal Random Structure** (calculated with REML)	**AIC**	**Step 1: Optimal Random Structure** (calculated with REML)	**AIC**
Day of life as random slope, fish ID as random intercept	-972.25	Day of life as random slope, fish ID as random intercept	-1263.81
Fish ID as random intercept	-647.03	Fish ID as random intercept	-333.83
No random structure	490.83	No random structure	1050.79
**Step 2: Optimal Fixed Structure** (calculated with Maximum Likelihood)		**Step 2: Optimal Fixed Structure** (calculated with Maximum Likelihood)	
log(**Growth**) ~ **Size** + **Temperature** + **Heatwave Class**	-2027.85	log(**Growth**) ~ **Size** x **Temperature** x **Heatwave Class**	-2550.42
log(**Growth**) ~ **Size** x **Temperature** x **Heatwave Class**	-2023.63	log(**Growth**) ~ **Size** x **Heatwave Class**	-2526.13
log(**Growth**) ~ **Size** x **Temperature**	-2022.67	log(**Growth**) ~ **Size** x **Temperature**	-2515.12
log(**Growth**) ~ **Temperature** x **Heatwave Class**	-1990.72	log(**Growth**) ~ **Size** + **Temperature** + **Heatwave Class**	-2511.67
log(**Growth**) ~ **Temperature**	-1977.84	log(**Growth**) ~ **Size**	-2487.84
log(**Growth**) ~ **Size**	-1966.2	log(**Growth**) ~ **Temperature** x **Heatwave Class**	-2439.45
log(**Growth**) ~ **Size** x **Heatwave Class**	-1965.59	log(**Growth**) ~ **Heatwave Class**	-2424.04
log(**Growth**) ~ **Heatwave Class**	-1919.38	log(**Growth**) ~ **Temperature**	-2364.94
log(**Growth**) ~ **Size** x **Temperature** x **Stomach Fullness** x **Heatwave Class**	-1291.20	log(**Growth**) ~ **Size** x **Temperature** x **Stomach Fullness** x **Heatwave Class**	-1662.79
log(**Growth**) ~ **Size** x **Temperature** x **NMS Axis 1 Score** x **Stomach Fullness** x **Heatwave Class**	-1272.07	log(**Growth**) ~ **Size** x **Temperature** x **NMS Axis 1 Score** x **Stomach Fullness** x **Heatwave Class**	-1612.71
log(**Growth**) ~ **Size** x **Temperature** x **NMS Axis 1 Score** x **Stomach Fullness** x **Abundance** x **Heatwave Class**	-1264.71	log(**Growth**) ~ **Size** x **Temperature** x **NMS Axis 1 Score** x **Stomach Fullness** x **Abundance** x **Heatwave Class**	-1588.65
**Step 3: Optimal Error Structure** (calculated with REML)		**Step 3: Optimal Error Structure** (calculated with REML)	
With AR1 autocorrelation error structure	-2256.34	With AR1 autocorrelation error structure	-2891.43
No error structure	-1997.58	No error structure	-2490.22

Table S7: Marginal mean effects for July and August relative growth (mm/mm/day) for best-fit models before, between, and during marine heatwaves. All values are adjusted to otolith increment number 11. This is the companion table for Figure 5 in the main text.

July				August			
**Before**							
	**Temperature**				**Temperature**		
Size	7.96	9.04	10.12	Size	8.95	10.27	11.59
26.7	--	--	--	41.8	--	--	--
32.5	0.016	0.014	0.013	50.2	0.019	0.014	0.011
38.2	0.015	0.013	0.012	58.7	0.015	0.012	0.010
44.0	0.014	0.013	0.011	67.1	0.012	0.011	0.009
49.7	0.013	0.012	0.011	75.6	0.010	0.009	0.009
55.5	0.012	0.011	0.010	84.1	0.008	0.008	0.008
61.3	0.012	0.011	0.009	92.5	0.006	0.007	0.008
67.0	0.011	0.010	0.009	101.0	0.005	0.006	0.007
72.8	--	--	--	109.4	--	--	--
78.5	--	--	--	117.9	--	--	--
84.3	--	--	--	126.4	--	--	--
90.1	--	--	--	134.8	--	--	--
95.8	--	--	--	140.0	--	--	--
**Between**							
	**Temperature**				**Temperature**		
Size	7.96	9.04	10.12	Size	8.95	10.27	11.59
26.7	--	--	--	41.8	--	--	--
32.5	--	--	--	50.2	--	--	--
38.2	0.016	0.014	0.013	58.7	0.009	0.009	0.010
44.0	0.015	0.013	0.013	67.1	0.008	0.009	0.010
49.7	0.014	0.013	0.012	75.6	0.007	0.008	0.009
55.5	0.013	0.012	0.011	84.1	0.007	0.007	0.008
61.3	0.013	0.011	0.011	92.5	0.006	0.007	0.008
67.0	0.012	0.011	0.010	101.0	0.006	0.006	0.007
72.8	0.011	0.010	0.009	109.4	0.005	0.006	0.006
78.5	0.011	0.009	0.008	117.9	0.005	0.005	0.006
84.3	0.010	0.009	0.008	126.4	0.005	0.005	0.006
90.1	0.009	0.008	0.007	134.8	--	--	--
95.8	0.009	0.008	0.007	140.0	--	--	--
**Heatwave**							
	**Temperature**				**Temperature**		
Size	7.96	9.04	10.12	Size	8.95	10.27	11.59
26.7	--	--	--	41.8	--	--	--
32.5	--	--	--	50.2	--	--	--
38.2	0.017	0.016	0.014	58.7	--	--	--
44.0	0.016	0.015	0.013	67.1	--	--	--
49.7	0.015	0.014	0.012	75.6	0.014	0.013	0.012
55.5	0.014	0.013	0.012	84.1	0.012	0.011	0.011
61.3	0.014	0.012	0.011	92.5	0.010	0.010	0.009
67.0	0.013	0.012	0.010	101.0	0.008	0.008	0.008
72.8	0.012	0.011	0.010	109.4	0.007	0.007	0.007
78.5	0.011	0.010	0.009	117.9	0.006	0.006	0.006
84.3	--	--	--	126.4	0.005	0.005	0.005
90.1	--	--	--	134.8	--	--	--
95.8	--	--	--	140.0	--	--	--
